# Control of brain patterning by Engrailed paracrine transfer: a new function of the Pbx interaction domain

**DOI:** 10.1242/dev.114181

**Published:** 2015-05-15

**Authors:** Christine Rampon, Carole Gauron, Thibault Lin, Francesca Meda, Edmond Dupont, Adrien Cosson, Eliane Ipendey, Alice Frerot, Isabelle Aujard, Thomas Le Saux, David Bensimon, Ludovic Jullien, Michel Volovitch, Sophie Vriz, Alain Joliot

**Affiliations:** 1 Université Paris Diderot, Sorbonne Paris Cité, Paris 75205, Cedex 13, France; 2 Center for Interdisciplinary Research in Biology (CIRB) - CNRS UMR 7241, INSERM U1050, Labex MemoLife, PSL Research University, Collège de France, Paris F-75005, France; 3 École Normale Supérieure, Institute of Biology at the Ecole Normale Supérieure (IBENS), CNRS UMR8197, INSERM U1024, PSL Research University, Paris F-75005, France; 4 Ecole Normale Supérieure-PSL Research University, Département de Chimie, UMR 8640 CNRS-ENS-UPMC PASTEUR, 24, rue Lhomond, Paris 75005, France; 5 Laboratoire de Physique Statistique, UMR CNRS-ENS 8550, Paris F-75005, France; 6 Department of Chemistry and Biochemistry, UCLA, Los Angeles, CA 90095-1569, USA

**Keywords:** Engrailed 2, Diencephalic-mesencephalic boundary, Homeoprotein signalling, Hexapeptide

## Abstract

Homeoproteins of the Engrailed family are involved in the patterning of mesencephalic boundaries through a mechanism classically ascribed to their transcriptional functions. In light of recent reports on the paracrine activity of homeoproteins, including Engrailed, we asked whether Engrailed intercellular transfer was also involved in brain patterning and boundary formation. Using time-controlled activation of Engrailed combined with tools that block its transfer, we show that the positioning of the diencephalic-mesencephalic boundary (DMB) requires Engrailed paracrine activity. Both zebrafish Eng2a and Eng2b are competent for intercellular transfer *in vivo*, but only extracellular endogenous Eng2b, and not Eng2a, participates in DMB positioning. In addition, disruption of the Pbx-interacting motif in Engrailed, known to strongly reduce the gain-of-function phenotype, also downregulates Engrailed transfer, thus revealing an unsuspected participation of the Pbx interaction domain in this pathway.

## Abstract (precis)

**SUMMARY:** The paracrine activity of Engrailed, together with its PBX-interacting hexapeptide motif, is involved in boundary formation during brain development in zebrafish.

## INTRODUCTION

Engrailed proteins form a subclass of the homeoprotein transcription factor family that play multiple roles in the patterning of metazoan embryos. They specify the posterior identity in the developing fly wing (Hidalgo, 1996), control the midbrain-hindbrain development and are required to pattern the optic tectum in vertebrates (Logan et al., 1996; Araki and Nakamura, 1999; Liu and Joyner, 2001; Scholpp and Brand, 2001; Scholpp et al., 2003). Conserved domains in Engrailed proteins include the homeodomain (HD, the DNA-binding motif that defines homeoproteins; Gehring et al., 1994) and a hexapeptide shared with many homeoproteins, which is responsible for the interaction with cofactors of the PBC (exd and Pbx) family (Peltenburg and Murre, 1996). In addition to their cell-autonomous action, homeoproteins can also transfer between cells through their secretion and internalisation, both relying on non-conventional routes (Volovitch et al., 1993; Prochiantz and Joliot, 2003; Spatazza et al., 2013a). The transfer property primarily resides within the HD itself, which contains two distinct peptide motifs involved in secretion and internalisation (Derossi et al., 1994; Dupont et al., 2007). This unusual behaviour for transcription factors endows several homeoproteins (which by definition all contain the HD motif) with paracrine signalling properties, as reported for Engrailed in fly crossvein development (Layalle et al., 2011) or vertebrate axonal retino-tectal projection (Brunet et al., 2005; Wizenmann et al., 2009), as well as for other homeoproteins in neuronal plasticity (Sugiyama et al., 2008; Beurdeley et al., 2012; Spatazza et al., 2013b), eye field development (Lesaffre et al., 2007), oligodendrocyte migration (Di Lullo et al., 2011) or cell proliferation (Zhou et al., 2012).

One of the best documented roles of Engrailed in vertebrates is its ability to regulate boundary formation during brain development (Joyner, 1996), classically referring to the (intracrine) transcriptional activity of the protein but without considering the possible contribution of its paracrine activity. To address this issue, we chose zebrafish because the formation of the diencephalic-mesencephalic boundary (DMB) has been elegantly analysed in this model by gain- and loss-of-function approaches. The two early expressed Engrailed proteins (Eng2a and Eng2b) are key determinants of forebrain identity at the DMB (Scholpp and Brand, 2001; Scholpp et al., 2003; Erickson et al., 2007). Because it has been shown that in mice, chicken and *Xenopus* axon guidance in the optic tectum is dependent on the paracrine activity of Engrailed (Brunet et al., 2005; Wizenmann et al., 2009), we asked whether diencephalon-mesencephalon patterning also depends on this activity. To study the role of Engrailed intercellular transfer in the establishment of the DMB, we took advantage of our ability to precisely control the temporal activity of proteins fused to the oestrogen receptor ligand-binding domain ER^T2^. Upon photoactivation, a caged tamoxifen analogue (cyclofen) binds to the ER^T2^ domain (Sinha et al., 2010a,b), releasing the fusion proteins from the complex they form with cytoplasmic chaperones. This approach, combined with the use of antibodies that block intercellular protein transfer *in vivo*, allowed us to address the role of Engrailed paracrine activity in brain patterning.

## RESULTS

### Engrailed gain of function reduces diencephalon size by paracrine signalling

**Fig. 1.**
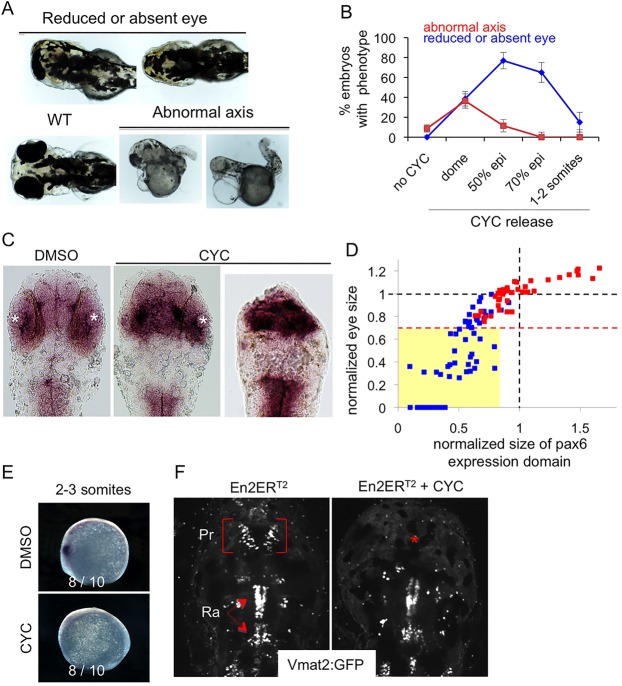
**Engrailed gain of function results in reduction in both *pax6* expression domain and eye size.** (A-F) mRNA encoding En2ER^T2^ was injected at the one-cell stage and the protein was activated at different times of development. Eye size reduction (up to total disappearance) and axis abnormality phenotypes (A) were scored at different times of activation controlled by cyclofen release (B). WT, wild type; epi, epiboly. Diencephalons were measured on flat-mount embryos as the anterior *pax6* expression domain (C) and compared to the eye phenotype (mean of the two eye sizes) for each embryo (D), demonstrating the correlation between these two parameters. White asterisks, eye position; red squares, control; blue squares, activated Engrailed. The dashed red line indicates the limit below which eyes were scored as reduced (or absent). All the embryos of this class (yellow box) have a small anterior *pax6* expression domain. Additional effects of En2 activation were apparent at the 2-3 somite stage with the loss of the anterior *pax6* expression domain (E) and at 4 dpf with the selective loss of the pretectal *vmat2*:GFP neuronal cluster (red asterisk) (F). Pr, pretectal cluster; Ra, raphe.

Injection of *eng2b* mRNA at the one-cell stage results in an anterior shift of the DMB (Scholpp et al., 2003). Given the multiple functions of Engrailed, we chose to perform inducible gain-of-function experiments by using the precisely controlled timing of Engrailed activation. Following injection of its mRNA at the one-cell stage, the protein of interest fused to ER^T2^ (Feil et al., 1997) was activated upon UV illumination of caged cyclofen ligand, added in the water bath. We first used an orthologous chicken Engrailed 2 (En2), similar to the zebrafish Engrailed 2 proteins (Eng2a and Eng2b), because its photoactivation has been well characterised in a previous study (Fournier et al., 2013). The amount of injected RNA was calibrated to produce less than 10% of diencephalon malformation in the absence of En2-ER^T2^ activation (whatever the readout). En2-ER^T2^ expressed in zebrafish embryos was activated at various stages of development by photorelease of cyclofen. Reduction of the expression domain of the Pax6 diencephalic marker at 1 day post-fertilisation (dpf) (Scholpp et al., 2003) and reduction of eye size (up to total disappearance) at 2 dpf (Ando et al., 2001) are two reported hallmarks of the En gain-of-function phenotype at the DMB. To determine the optimal time window, we first concentrated on eye size for simplicity. En2-ER^T2^ photo-activation prior to gastrulation induced a widespread insult resulting in abnormal axis and heart development defects in 40% of the embryos, whereas En2-ER^T2^ photoactivation at 50% and 70% epiboly impaired eye formation without any sign of axis abnormality (Fig. 1A,B). Activation of En2-ER^T2^ at the beginning of somitogenesis (1-2 somites) induced almost no phenotype (Fig. 1B). Activation at the 50-70% epiboly time window was thus used for all of the following experiments unless specified.

We next performed a detailed quantitative analysis of the phenotype induced by En activation (the sample size of all experiments is summarised in supplementary material Table S1), by measuring for each embryo the size of the eye field (mean of the two eyes) and the size of the diencephalon, as revealed by the anterior expression domain of *pax6* (*pax6a*) at 1 dpf (Fig. 1C,D). The size of the diencephalon was significantly reduced (*P*<0.001) upon En2 activation at 50-70% epiboly and strongly correlated (*R*=0.92) with a corresponding reduction of the eye size (Fig. 1D). Visual scoring of the eye phenotype (performed in a double-blind manner), which perfectly fit with eye measurement (Fig. 1D), was used as readout of diencephalon shortening in subsequent experiments. Additional phenotypes associated with En2 gain of function were observed at other stages of development, from the inhibition of *pax6* expression at the 2-3 somite stage (Fig. 1E), to the selective loss of the pretectal (diencephalic) neural cluster of *ETvmat2*:GFP (*vmat2* is also known as *slc18a2* – ZFIN) neurons at 4 dpf (Wen et al., 2008) (Fig. 1F).

**Fig. 2.**
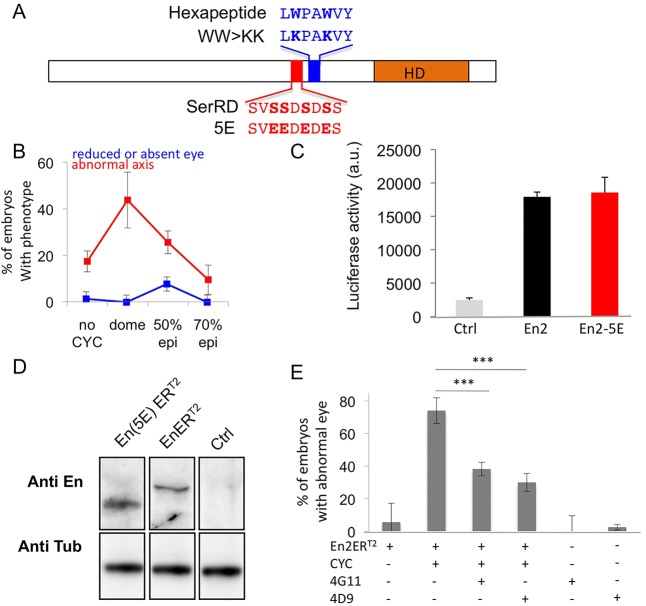
**Engrailed gain of function involves the paracrine signalling properties of En.** (A) Description of the two En2 mutants defective for paracrine signalling. (B) Diencephalon shortening requires En2 transfer. Activation of the En2(5E) transfer-deficient mutant following mRNA injection did not affect the size of the diencephalon. epi, epiboly. (C) Transcriptional activity of wild-type En2 and En2(5E). *MAP1B*:luciferase reporter plasmid was transfected into HeLa cells with an empty vector (ctrl) or together with the indicated constructs and analysed for luciferase activity after 24 h. a.u., arbitrary units. (D) *In vivo* ectopic expression of En proteins. Zebrafish embryos were injected at the one-cell stage with the indicated constructs. Following CYC addition at 50% epiboly, cell extracts from 90% epiboly embryos were prepared and analysed by western blotting with polyclonal (anti-En) or monoclonal (anti-tub) antibodies. (E) Inhibition of En2 transfer rescued the phenotype of En2 activation. Zebrafish embryos were injected at the one-cell stage with mRNA encoding En2ER^T2^ and injected again in the extracellular space at the blastula stage with two different anti-En antibodies. CYC was added at 50% epiboly in the water bath to activate the protein. Eye phenotypes were scored at 2 dpf. The error bars represent statistical errors. ***P<0.001.

Specific motifs within Engrailed regulate the transfer of the protein, either through its secretion or its internalisation (Joliot et al., 1998; Maizel et al., 1999, 2002) (Fig. 2A), and En2 paracrine signalling activity is lost upon mutation of the internalisation motif (Brunet et al., 2005). A mutation preventing secretion, En2(5E)-ER^T2^ (Maizel et al., 2002), was introduced in the sequence of En2-ER^T2^, and its impact on Engrailed activity was analysed in the gain-of-function assay described above (Fig. 1). Activation of either mutated protein at the dome stage induced abnormal heart and axis development in ∼40% of the embryos (Fig. 2B), indicating that this widespread effect did not involve Engrailed 2 intercellular transfer. By contrast, En2(5E)-ER^T2^ was not able to induce an eye phenotype when photo-activated at 70% epiboly (Fig. 2B), suggesting that intercellular transfer of Engrailed 2 is involved in brain patterning. Besides the reported effect of this mutation on the transfer process, we could not exclude that other functions could be affected as well, including at the transcriptional level. To test this hypothesis, the transcriptional activities of Engrailed and of its mutated form were compared on the *Map1b* promoter fused to a luciferase reporter in HeLa cells (Fig. 2C). A 2 kilobase long fragment of the *Map1b* rat promoter is regulated by Engrailed in cell culture and *in vivo* in vertebrates (Montesinos et al., 2001). En2 and En2(5E) were equally competent for MAP1B activation, increasing luciferase expression by sevenfold. We also verified the expression of the two proteins in zebrafish. Following RNA injection at the one-cell stage and cyclofen activation, cell extracts from injected embryos expressing En2-ER^T2^ or the mutant were analysed by western blotting with a polyclonal anti-Engrailed antibody (Fig. 2D). En2-ER^T2^ and En2(5E)-ER^T2^ were expressed at similar levels.

In zebrafish, Lesaffre et al. have originally demonstrated that extracellular injection of antibodies directed against homeoproteins is an efficient tool for blocking homeoprotein paracrine activity, leaving the intracrine activity intact (Lesaffre et al., 2007). We applied this strategy to rescue the gain-of-function phenotype induced by En2 ectopic expression. Zebrafish embryos were injected at the one-cell stage with *en2-ER^T2^* mRNA followed at blastula stage by injection, in the intercellular space, of two different monoclonal antibodies directed against either the homeodomain (4D9) or an N-terminal motif (4G11) of Engrailed (Patel et al., 1989; Ericson et al., 1997). Cyclofen or buffer alone was added at 50% epiboly and eye phenotypes were scored at 2 dpf (Fig. 2E). Both 4D9 and 4G11 injections were able to rescue the eye phenotype induced by En2 gain of function, thus corroborating the requirement for En2 intercellular transfer and paracrine activity. Antibodies injected in the intercellular space at the blastula stage do not enter into cells, at least up to the shield stage (Lesaffre et al., 2007), and thus do not perturb homeoprotein intracrine actions. To confirm this observation in our own experimental set-up, which targets slightly later embryonic stages, FITC-labelled anti-Engrailed antibodies were injected using the same protocol and labelling was analysed up to the 90% epiboly stage in live animals. FITC staining remained almost exclusively sequestered in the intercellular space and could only be detected in few pinocytic vesicles at the later time-point, but never within nuclei (supplementary material Fig. S1).

### Zebrafish Engrailed 2a and 2b are competent for intercellular transfer *ex vivo* and *in vivo*

**Fig. 3.**
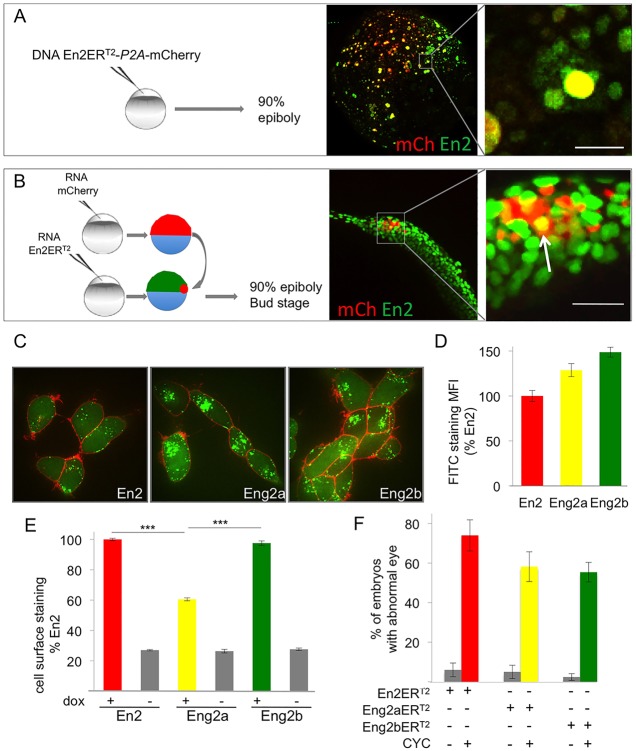
**Zebrafish Engrailed 2a and 2b are able to transfer between cells *in vivo* and *ex vivo*.** (A,B) *In vivo* intercellular transfer of En2 protein. (A) The En2ER^T2^-P2A-mCherry plasmid was injected at the one-cell stage (10 ng/µl). Embryos were fixed at 90% epiboly and immunostained for En2ER^T2^ (green) and mCherry (red). En2 transfer was visualised by the presence of En2ER^T2^ staining (green) in non-injected cells (negative for mCherry staining). (B) Cells expressing mCherry (red) were transplanted into 30% epiboly embryos ubiquitously expressing En2ER^T2^. En2ER^T2^ transfer was revealed by the detection of EnER^T2^ signal (green) in mCherry-positive cells (yellow cells, arrow). Scale bars: 20 µm. (C,D) Internalisation of Eng2a and 2b in HEK293 cells. Following extracellular addition, internalisation of fluorescein-labelled En2, Eng2a or Eng2b protein (green) was visualised (C) and quantified (D) after 1 h incubation at 37°C in the presence of 0.4% Trypan Blue (red) to quench extracellular fluorescence. MFI, mean fluorescence intensity. (E) Secretion of Eng2a and 2b. HEK293 cells expressing the indicated proteins under the control of doxycycline were cultured for 24 h and cell surface accumulation of the secreted protein was monitored by flow cytometry. ***P<0.001. (F) Paracrine activity of Eng2a and Eng2b proteins. mRNA encoding En2ER^T2^, Eng2aER^T2^ or Eng2bER^T2^ were injected at the one-cell stage, the protein was activated with CYC at 50% epiboly and eye defects were scored at 30 hpf. The error bars represent statistical errors (F) or s.e.m. (D,E).

Homeoprotein paracrine activity cannot be dissociated from its ability to transfer between cells, thus it was crucial to confirm the actual intercellular transfer of the inducible form of En2-ER^T2^ in the fish. We used two complementary approaches to directly visualise the transfer process *in vivo*. In the first one, En2-ER^T2^-expressing cells were specifically labelled by tandem translation of mCherry from the same mRNA molecule (En2-ER^T2^-P2A-mCherry). Injection of plasmid DNA at the one-cell stage led to mosaic expression of the injected construct (encoding both mCherry and En2-ER^T2^), as classically reported. Embryos treated with cyclofen at 50% epiboly to activate En2-ER^T2^ were fixed at 90% epiboly and processed for immunodetection of En2-ER^T2^ and mCherry. En2-ER^T2^ was detected in non-expressing cells characterised by the absence of mCherry staining, suggesting En2-ER^T2^ intercellular transfer (Fig. 3A; supplementary material Fig. S2 for separate channels). In the same experimental set-up, following injection of a plasmid DNA expressing the mutated protein En2(5E)-ER^T2^-P2A-mCherry, the *in vivo* intercellular transfer of the mutated form of En2 was drastically reduced (supplementary material Fig. S2). To unambiguously confirm *in vivo* intercellular transfer of En2 with the reverse strategy, mCherry-expressing cells were grafted into En2-ER^T2^-expressing embryos (Fig. 3B). Cells co-labelled with mCherry and En2-ER^T2^ were detected (Fig. 3B). This unambiguously demonstrates the intercellular transfer of En2-ER^T2^ between 50% and 90% epiboly, at the time of its paracrine action on brain patterning.

Zebrafish Engrailed (Eng2a and Eng2b) differ to some extent from chicken Engrailed (En2) outside the highly conserved homedomain. We asked whether they retained the intercellular transfer property. We compared the secretion and internalisation of the two zebrafish proteins to those of En2. Homeoprotein internalisation was directly visualised in cultured live cells, following incubation of the fluorescently labelled recombinant proteins in the medium. Importantly, the cell-non-permeable fluorescence quencher Trypan Blue was added to the medium just prior to imaging in order to block extracellular fluorescence signal while leaving the intracellular one intact. As shown in Fig. 3C,D, all three proteins were similarly internalised, although internalisation was more efficient for Eng2b.

In cell culture, secreted homeoproteins mainly concentrate at the cell surface owing to strong electrostatic interactions with carbohydrates, and they can be detected on live cells with conventional flow cytometry techniques, as reported for FGF2 secretion (Engling et al., 2002). When expressed in the human embryonic kidney (HEK)293-FlpIn TREX cell line (which allows inducible expression upon doxycycline addition), all three proteins were detected at the cell surface (Fig. 3E), and thus were efficiently secreted, although at lower levels for Eng2a. These experiments demonstrate that *ex vivo*, intercellular transfer is a shared property of the three Engrailed proteins. To confirm these results *in vivo*, we verified that Eng2a and 2b behaved similarly to En2 in the transfer assay set-up in the fish (supplementary material Fig. S2).

We then compared the efficiency of Eng2a, Eng2b and En2 in inducing the eye phenotype described above. Following injection of mRNA encoding En2-ER^T2^, Eng2a-ER^T2^ or Eng2b-ER^T2^ and activation by cyclofen, phenotypes were scored at 30 h post fertilisation (hpf) by counting the number of embryos with eye defects. All three proteins were able to induce an eye phenotype, with comparable efficiencies (Fig. 3F).

### Intercellular transfer of Eng2b is required for normal brain patterning

**Fig. 4.**
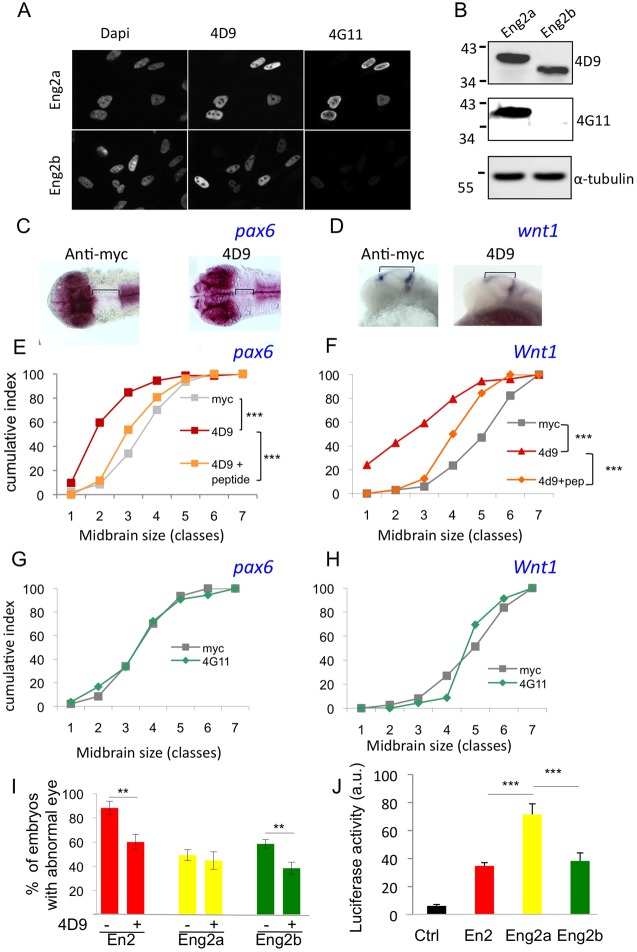
**Intercellular transfer of Eng2b but not 2a is involved in DMB positioning.** (A,B) Co-detection of Eng2 proteins with FITC-labelled 4D9 and TRITC-labelled 4G11 antibodies by immunohistochemistry (A) or western blot analysis on HeLa cells (B). Using both techniques, 4D9 recognised Eng2a and Eng2b, whereas 4G11 recognised only Eng2a. (C-H) Paracrine activity of endogenous En2 proteins. Zebrafish embryos were injected in the intercellular space at blastula stage with anti-myc or anti-Engrailed (4G11 or 4D9) antibody. Mesencephalon length was quantified by *pax6* (C,E,G) or *wnt1* (D,F,H) *in situ* hybridisation. Representative *in situ* hybridisation staining of *pax6* (dorsal view) (C) and *wnt1* (lateral view) (D). Measurements were distributed into seven size classes (smallest size, class 1) and plotted as cumulative frequency index. 4D9 extracellular injection induced mesencephalon shortening (E,F) in a dose-dependent manner (supplementary material Fig. S4), which was the case for neither anti-myc (E-H) nor 4G11 injection (G,H). Co-injection of the 4D9 antibody with its epitope peptide (pep) significantly reduced this effect (E,F). ***P<0.0001, Mann–Whitney tests. (I) Inhibition of homeoprotein transfer rescued the phenotype of Eng2b activation but not of Eng2a. Zebrafish embryos were injected at the one-cell stage with the indicated mRNAs and injected again in the extracellular space at the blastula stage with 4D9 anti-En antibody. CYC was added at 50% epiboly in the water bath to activate the protein. Eye phenotypes were scored at 2 dpf. The error bars represent statistical errors. (J) Transcriptional activity of En2 and the two zebrafish Eng2. The *MAP1B*:luciferase reporter plasmid was transfected into HeLa cells along with an empty vector (Ctrl) or together with the indicated constructs, and cells were analysed for luciferase activity after 24 h. a.u., arbitrary units. **P<0.01, ***P<0.001.

In the course of the *ex vivo* experiments, we noticed that the two antibodies, 4D9 and 4G11, behaved differently on the two zebrafish Engrailed 2 proteins. Although 4D9 recognised both Eng2a and Eng2b, 4G11 recognised only Eng2a on western blots and by immunohistochemistry (Fig. 4A,B). This observation offered us the opportunity to address the role of the paracrine activity of each of the two endogenous Eng2 proteins in normal brain patterning. Zebrafish embryos were injected into the intercellular space at the blastula stage with either 4G11, 4D9 or control (anti-myc) antibodies. Embryos were fixed at 24 hpf, and the size of the mesencephalon was measured by *in situ* hybridisation against *pax6* or *wnt1*, which delineate the midbrain (Fig. 4C,D). Upon injection, 4D9 (recognising both Eng2a and Eng2b) reduced the size of the mesencephalon (Fig. 4E,F, in a dose-dependent manner; supplementary material Fig. S4), whereas 4G11 (recognising only Eng2a) had no effect (Fig. 4G,H). To confirm the specificity of action of 4D9, we verified that the effect induced by 4D9 injection was abolished upon pre-incubation with its cognate epitope peptide (Fig. 4E,F). It is worth noting that 4D9 in this assay had no effect on eye size (supplementary material Fig. S5), rhombomere formation or oligodendrocyte migration (supplementary material Fig. S6), corroborating the specificity of its action on anterior brain patterning. Given that 4D9 and 4G11 were both able to rescue the reduced eye phenotype induced by En2 activation (Fig. 2E), the absence of the phenotype in 4G11-treated embryos cannot be simply attributed to its inability to block intercellular transfer. Although both Eng2a and Eng2b have the capacity to transfer between cells, only Eng2b paracrine signalling activity is required for correct brain patterning. This result led us to reconsider the gain-of-function phenotypes induced by the two zebrafish Engrailed proteins and, more precisely, their sensitivity to blocking antibody treatment. Surprisingly, treatment with 4D9, which equally recognises Eng2a and b (Fig. 4A,B), decreased the frequency of eye phenotypes induced by Eng2b but not by Eng2a mRNA injection (Fig. 4I). If Eng2a paracrine action did not account for the eye phenotype, we asked whether the intracrine action of the protein differs from that of Eng2b or En2. The transcriptional activity of the Eng2a tested on the MAP promoter in HeLa cells was increased almost twofold compared with that of the other proteins (Fig. 4J), suggesting a more efficient intracrine action of Eng2a.

**Fig. 5.**
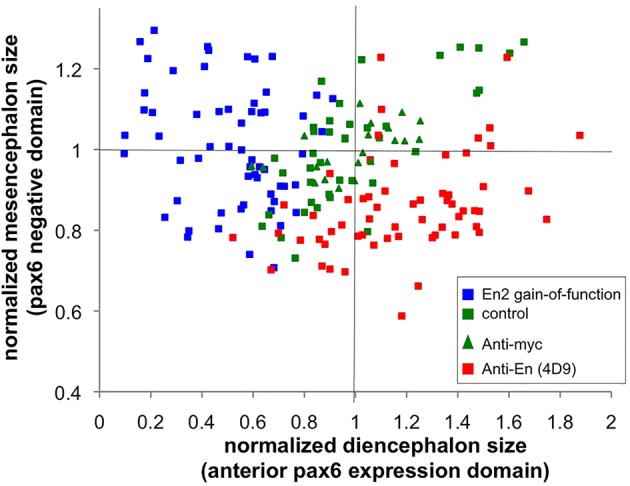
**Relative size of the anterior *pax6*-positive domain and *pax6*-negative domain.** Sizes of the anterior *pax6* expression domain and of the *pax6*-negative domain were measured on flat-mount embryos stained for *pax6* expression by *in situ* hybridisation at 1 dpf. Green and blue squares, En2 mRNA was injected at the one-cell stage and CYC was added (blue) or not (green) at 50% epiboly. Green triangles (anti-myc control) and red squares (anti-Engrailed), antibodies were injected at the blastula stage to block Engrailed paracrine activity.

It has been proposed previously that Engrailed gain of function induced a transformation of forebrain tissue identity towards a mesencephalic fate rather than an intrinsic shortening of the diencephalon (Scholpp et al., 2003). We indeed observed that activation of En2 at the appropriate time did not affect apoptosis during development (supplementary material Fig. S7), supporting an identity switch between the two adjacent structures. To confirm this hypothesis, we compared the relative size of the anterior *pax6*-positive (diencephalic) and adjacent *pax6*-negative (mesencephalic) domains in all control and treated animals (injected with 4D9 antibody) previously analysed in Fig. 4. Mesencephalon shortening induced by blocking En2 transfer with 4D9 (red squares) was correlated with a corresponding enlargement of the diencephalon (Fig. 5). To our surprise, the reverse effect in gain-of-function experiments was not as clear, the size of the mesencephalon being not statistically affected by En2 ectopic expression, despite the significant shortening of the diencephalon (blue squares) (Fig. 5).

### The Pbx interaction domain regulates Engrailed 2 paracrine activity

The effect of Eng2a on DMB positioning requires its interaction with Pbx co-factors (Erickson et al., 2007). The two tryptophan residues located within the Pbx interaction domain of Eng2a (or ‘hexapeptide’, Fig. 2A) are mandatory in mediating the cooperative binding of the complex on DNA, and their mutation into lysine drastically reduces the gain-of-function phenotype induced by Eng2a overexpression (Erickson et al., 2007). These results were consistent with the cell-autonomous initial activation of Eng2b transcription by the Eng2a-Pbx complex (Scholpp and Brand, 2001; Erickson et al., 2007), but did not reveal the possible connection, if any, with the paracrine activity of Engrailed.

**Fig. 6.**
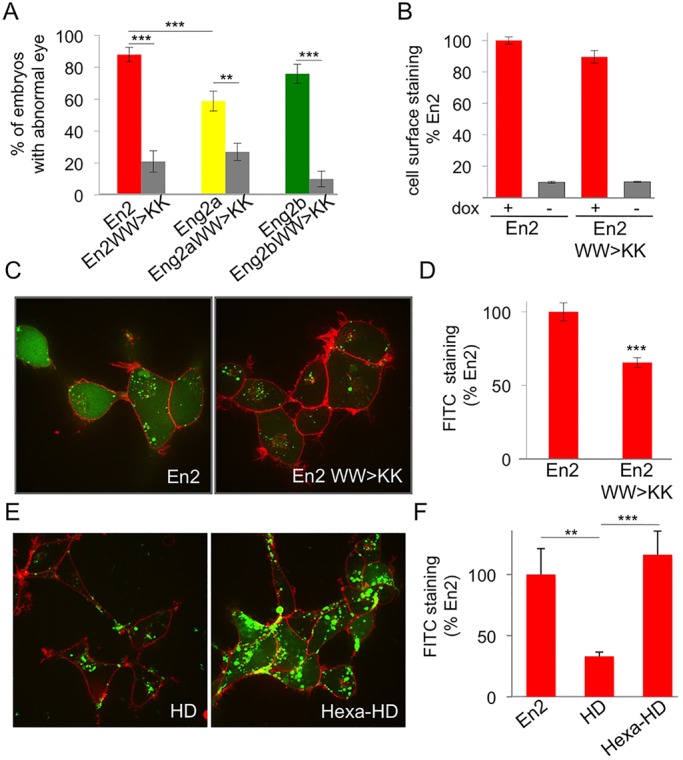
**Pbx interaction domain controls En paracrine activity.** (A) Phenotypic analysis of hexapeptide-mutated forms of En2 protein. Compared to their wild-type counterparts, the eye phenotype induced by the activation of hexapeptide mutants was drastically reduced. The error bars represent statistical errors. (B-D) Intercellular transfer of En2 hexapeptide mutant *ex vivo.* HEK293 cells expressing the indicated proteins under the control of doxycycline were cultured for 24 h and cell surface accumulation of the secreted protein was monitored by flow cytometry (B). Internalisation of fluorescein-labelled En2, or En2 WW>KK (green) was visualised (C) and quantified (D) after 1 h incubation at 37°C in the presence of 0.4% Trypan Blue (red) to quench extracellular fluorescence. (E,F) Internalisation of fluorescein-labelled En2, En2HD, Hexa-En2HD was visualised (E) and quantified (F) as described above. The error bars represent statistical errors (A) or the s.e.m. (B,D,F). **P<0.01, ***P<0.001.

We first assessed the selectivity of the hexapeptide mutation towards Eng2a function by engineering equivalent WW>KK substitutions in the En2, Eng2a and Eng2b constructs. The mRNAs coding for the mutant forms of Engrailed-ER^T2^ were injected into zebrafish embryos at the one-cell stage, embryos were incubated in cyclofen at 50% epiboly, and eye defects were scored at 30 hpf. Introducing the WW>KK double mutation similarly decreased the occurrence of the eye phenotype for the three Engrailed 2 proteins (Fig. 6A). Although the mutation might appear to selectively affect the intracrine component of Engrailed action, we wondered whether the removal of two tryptophans near the homeodomain might also affect intercellular transfer, given the crucial role of such residues in the internalisation process (Le Roux et al., 1993; Derossi et al., 1994). We tested the ability of the mutant forms of En2 to exit and enter cells using the cell culture quantitative assays described above. When expressed in HEK cells, the mutant and wild-type proteins were secreted to similar extents (Fig. 6B), meaning that the integrity of the hexapeptide is not required for En2 secretion. By contrast, the internalisation of the mutated protein by HEK cells was significantly reduced compared with that of its wild-type counterpart (Fig. 6C,D). We reasoned that, conversely, the presence of the hexapeptide on its own might increase the uptake process. This would corroborate unpublished observations that in this experimental model, full-length En2 homeoprotein uptake was higher than that of an En2 homeodomain fragment (Fig. 6C,E). A 12 amino-acid long peptide bearing the hexapeptide was fused to the N-terminus of the En2 homeodomain and its uptake was evaluated. Addition of the hexapeptide restored the uptake of the En2 homeodomain to levels comparable to those of the full-length homeoprotein (Fig. 6C,E,F). Overexpression of Pbx neither significantly alters En2 location nor interferes with its intercellular transfer (secretion and internalisation) properties (supplementary material Fig. S3), although internalisation was occasionally slightly reduced. We then conclude that the hexapeptide per se potentiates homeodomain uptake and, therefore, that the WW>KK mutation, known to disrupt Pbx-Engrailed transcriptional complexes, also impairs the paracrine component of Engrailed action, by lowering the efficacy of protein trafficking.

## DISCUSSION

In this study, we show that ectopic En2 modifies brain patterning only when activated between dome and 1-2 somite stages, corroborating and refining the initial experiments performed with Eng2a (Ando et al., 2001) or Eng2b (Scholpp et al., 2003). It is worth noting that this coincides with the time that Eng2a and b are expressed (Scholpp et al., 2003), suggesting that the time window during which Engrailed controls anterior brain patterning is relatively narrow (90% epiboly to 1-2 somite stages). Most importantly, we demonstrate that the paracrine activity of En2 homeoprotein is required for this process, using two distinct but complementary strategies. First, brain patterning defects are not observed with the secretion-defective mutant En2(5E), although its ability to regulate transcription remains unaffected. Secondly, these defects are reversed by the selective inhibition of En2 paracrine action through the extracellular injection of two different anti-En antibodies. By contrast, axis malformations resulting from early En2 activation are equally observed with wild-type and mutant proteins and are insensitive to antibody treatment. Indeed, these axis malformations are likely to rely on unspecific activity.

During zebrafish development, *eng2a* is expressed slightly before *eng2b* and activates *eng2b* expression (Scholpp and Brand, 2001). However, the two Eng2 proteins behave differently regarding their paracrine activity, although both are competent for secretion and internalisation. The function of endogenous Eng2a relies preferentially on its intracrine action, because treatment with the 4G11 antibody, which recognises only Eng2a, has no effect on brain patterning. Moreover, 4D9 antibody, which recognises an invariant epitope within the three tested Engrailed proteins (supplementary material Fig. S8), antagonises the action of Eng2b and En2, but not of Eng2a. Although both are competent for intercellular transfer *ex vivo*, Eng2a and b, arising from a recent duplication, might have retained distinct modes of action (intracrine or paracrine), supported by the increased transcriptional activity of Eng2a. This enhanced intracrine activity might account for the eye phenotype in the context of ectopic expression where the protein is expressed throughout the embryo.

It should be noted that at 90% epiboly (when the paracrine action is required) *eng2a* expression occurs within the *eng2b* domain (Lun and Brand, 1998; Postlethwait et al., 1998). The broader expression of *eng2b* over *eng2a*, meaning that *eng2b* extends closer to the DMB, might account for the preferential requirement of Eng2b paracrine activity. Indeed, we have previously shown that extracellular Engrailed in the *Drosophila* wing disc has a limited diffusion, restricted to a few cell rows (Layalle et al., 2011). Similarly, the source of extracellular Engrailed in zebrafish might be restricted to the Eng2b-positive but Eng2a-negative cell population. Alternatively, we cannot exclude that endogenous Eng2a exerts some paracrine activity, but its loss would be compensated for by Eng2b paracrine activity. Such functional redundancy between the two Eng2 proteins has been observed in loss-of-function experiments, where the downregulation of either Eng2a or 2b by morpholino injection induced little phenotype compared to the co-injection of the two morpholinos (Scholpp et al., 2003).

Homeoprotein expression is often endowed with positional information. The sharp spatial restriction of the extracellular secreted protein from the edges of these territories combined with the requirement for intracrine (transcriptional or translational) homeoprotein action might be the two crucial spatial determinants that convert this positional information into the formation of actual boundaries with unique properties. However, the careful quantification of gain- and loss-of-function phenotypes leads to intriguing observations. According to the classical view of DMB positioning, the DMB shift inversely affects the two structures (mesencephalon and diencephalon) located at either side of the boundary, the expansion of one structure occurring at the expense of the other one. Quantification of the lengths of diencephalic and mesencephalic structures for each embryo, measured with the anterior *pax6* expression domain and the posterior adjacent *pax6*-negative domain, respectively, highlighted an inverted correlation between the size of the two structures in loss-of-paracrine-function experiments, but not in gain-of-function experiments. However, even in the latter case, the diencephalic identity is clearly affected, illustrated by the loss of pretectal *vmat2* neurons. Our attempts to quantify cell proliferation (by phospho-H3 staining) in the presence or absence of En2 activation were not conclusive, owing to high spatial heterogeneity at the level of the whole embryo and the fact that identification of the prospective anterior brain structures at this embryonic stage is still challenging. A likely hypothesis would be that, superimposed on DMB positioning, the relative growth of the two structures is regulated independently by other mechanisms, as proposed previously (Scholpp et al., 2003), which would be insensitive to Engrailed paracrine action.

In gain-of-function experiments, DMB positioning is sensitive to Engrailed paracrine activity, although one-cell stage Engrailed mRNA injection should lead to the broad expression of Engrailed, including in cells that respond to extracellular Engrailed. This suggests complementary functions of intracrine and paracrine activities displayed by the same homeoprotein. Such a situation appears to be a recurrent feature of homeoprotein dual activity, first described with the Ephrin signalling pathway in retinal ganglion cell (RGC) axonal guidance (Wizenmann et al., 2009), and more recently with the Dpp pathway in crossvein formation of the *Drosophila* wing (Layalle et al., 2011). In the latter case, the transcription of *dpp* is directly downregulated by Engrailed but, in the same cells, Engrailed transfer stimulates Dpp signalling (pMAD phosphorylation). The fact that ectopic expression of two analogous proteins (Eng2a and b) led to a similar eye phenotype through either intracrine or paracrine action reinforces the functional link between the two modes of homeoprotein action towards the same goal, a situation that has been reported for other proteins (Radisky et al., 2009).

The dual function of the hexapeptide is a new paradigm of the crosstalk between intracrine and paracrine homeoprotein action. This conserved motif is found in a subset of homeoproteins and governs the physical interaction with the PBC family of co-factors (Chang et al., 1995; Neuteboom et al., 1995; Phelan et al., 1995), although hexapeptide-independent complexes have been described recently (Hudry et al., 2012). Moreover, PBC proteins are crucial regulators of brain patterning at the DMB, acting in synergy with Eng proteins (Erickson et al., 2007). We found no evidence for a regulation of Engrailed transfer by PBC proteins. Pbx1 over-expression affected neither Engrailed secretion in HEK cells nor (or only slightly) Engrailed uptake. We propose that intracrine and paracrine actions of Engrailed cooperate for brain patterning and both actions depend on the hexapeptide through distinct mechanisms. The precise role of the hexapeptide in paracrine activity remains to be determined and, at this stage, we cannot formally exclude that En-Pbx interaction has some impact on Engrailed transfer. An extended mutation analysis encompassing the hexapeptide of Engrailed might help to discriminate between the two functions of this domain and evaluate the actual role of Pbx. It should be kept in mind that in addition to Engrailed proteins, all the Hox proteins of the paralog groups 1-8 contain the hexapeptide (Knoepfler and Kamps, 1995), mutation of which almost invariably leads to strong loss-of-function phenotype (Moens and Selleri, 2006). Our findings shed new light on the putative roles of this sequence as we show that its mutation impairs intercellular transfer, a shared property of many homeoproteins. This might lead to the revisiting of experiments in which mutation or lure (competing or decoy peptide) was assumed to act selectively on homeoprotein-PBC interactions without also considering paracrine homeoprotein signalling. This might be of importance in developmental processes and also in pathological contexts such as oncogenesis, given that the hexapeptide motif is the target of several therapeutic strategies (Morgan et al., 2007; Delval et al., 2011).

## MATERIALS AND METHODS

### Fish care

Zebrafish were maintained and staged according to Westerfield (1993). Experiments were performed using the standard Ab wild-type strain. The following zebrafish transgenic line was used: Tg(*ETvmat2*:GFP) (Wen et al., 2008). This transgenic line was a gift from Shuo Lin (UCLA, CA, USA). The embryos were incubated at 28°C. Developmental stages were determined as hours post-fertilisation (hpf). The animal facility obtained a French agreement from the ministry of agriculture for all the experiments performed in this study (agreement number C 75-05-12).

### Nucleic acids and antibody injection

Plasmids (10 ng/μl) were injected at the one-cell stage. *Engrailed-ER^T2^* or *mCherry* mRNA synthesis was performed using the mMESSAGE mMACHINE Transcription Kit from Ambion. Equivalent volumes of 30 ng/µl *engrailed-ER^T2^* mRNA or 50 ng/µl *mCherry* mRNA were injected into one-cell stage embryos. Mouse monoclonal antibodies (anti-Engrailed 4D9, developed by C. Goodman, and anti-Engrailed 4G11, developed by T. Jessell, both obtained from DSHB, University of Iowa, and anti-myc 9E10, Roche Applied Science) were used for *in vivo* injection (100 ng/µl) at the blastula stage as described previously (Lesaffre et al., 2007).

### Engrailed-ER^T2^ activation

Caged 4-OH cyclofen (cCYC) or 4-OH cyclofen (CYC) were prepared as described previously (Sinha et al., 2010a). Embryos injected with *engrailed-ER^T2^* mRNA at the one-cell stage were incubated in 3 µM cCYC and illuminated at specific time-points to determine the time window for Engrailed involvement in DMB position. For all other experiments involving Engrailed-ER^T2^ activation, 1 µM CYC was added in water at 50% epiboly as described previously (Sinha et al., 2010b).

### Immunohistochemistry

#### Whole-mount immunohistochemistry

Alexa Fluor 488-conjugated goat anti-mouse IgG (Life Technologies) was diluted 1:500, mouse anti-myc antibody (9E10, Roche) was diluted 1:500 and mouse anti-mCherry (Clontech) was diluted 1:250.

#### Cell culture immunocytochemistry

Cells grown on coverslips were fixed with paraformaldehyde (4%, 10 min, room temperature) in PBS, permeabilised with Triton X-100 (0.3%, 5 min, room temperature) and saturated with PBS containing 10% sheep serum before incubation with primary antibodies (4D9 or 4G11 anti-Engrailed antibody at 5 µg/ml, 1 h, room temperature) and secondary antibodies (Alexa Fluor 488 goat anti-mouse IgG, 1:500, 1 h, room temperature).

### *In situ* hybridisation and morphometric analysis

RNA probes and whole-mount *in situ* hybridisation were carried out using a robot (Intavis) according to standard procedure (Thisse and Thisse, 2008); details concerning the program used are available upon request. Morphometric analysis was performed on flat-mount 24 hpf embryos using Axiovision 4.8 software.

### Embryo imaging

Images were obtained using a Nikon microscope with a spinning-disk (Roper) operated with Metamorph premier 7.6 software, a Leica SP5 confocal microscope driven by LAS AF software or a binocular Nikon SMZ 1500.

### Grafting experiments

*mCherry* mRNA (50 ng/µl) was injected at the one-cell stage into donor embryos whereas host embryos were injected at the one-cell stage with *En2ER^T2^* mRNA (30 ng/µl). At the dome stage, 5-20 donor (red) cells were grafted to the animal pole of host embryos (Ho and Kimmel, 1993). Embryos were then cultured in 0.5% methylcellulose with 10 U/ml penicillin and 10 μg/ml streptomycin.

### Western blotting

#### Cell extracts

At 24 h after transfection, cells were washed three times (PBS), collected (PBS, 2 mM EDTA) and pelleted by centrifugation. The cell pellet was resuspended and sonicated before addition of Laemmli buffer and SDS-PAGE separation. Proteins were transferred to an immobilon membrane (GE Healthcare) and processed for western blot detection using horseradish peroxidase (HRP)-labelled secondary antibodies, chemiluminescence (ECL Plus, GE Healthcare) and detection (Fuji LAS 4000).

#### Embryos

Embryos (20 per sample) were kept dried at −80°C before resuspension in PBS supplemented with protease inhibitors and nuclease. Each sample was sonicated (Bioruptor, Diagenode) twice (5 min, 50% cycle, high intensity) and subjected to acetone precipitation. Protein pellets were resuspended in Laemmli buffer, quantified and processed for western blotting.

### Cell culture and transfection

Cell culture experiments were performed on COS, HeLa or HEK293 cells as specified in the text or legends, grown in DMEM supplemented with 10% fetal bovine serum. Transient transfections were performed with Lipofectamine 2000 (Life Technologies) according to the manufacturer’s instructions. Cells were cultured for an additional 24 h before being processed for analysis by FACS, western blotting, immunocytochemistry or transcription assay. The HEK293 cell line constitutively expresses the tetracycline repressor (HEK293-FlpIN/TREX, Life Technologies) and doxycycline was needed to induce protein expression when tetracycline-sensitive expression plasmids were transfected.

For transcription assays, HeLa cells were co-transfected with luciferase reporter together with the expression plasmids encoding the indicated proteins at a 1:3 ratio. At 24 h after transfection, cells were washed with PBS and lysed in PLB buffer (Promega). Luciferase activity was measured in triplicate on a 96-well plate luminometer (Tristar, Berthold) with the luciferase reporter assay kit (Promega).

### Internalisation

Cells (HEK293, 20,000 per well) were plated on µ-slide eight-well plates (Ibidi). After 24 h, the medium was removed and cells were incubated with the fluorescent protein (500 nM) diluted in DMEM without serum for 1 h at 37°C before visualisation on a spinning-disk microscope equipped with a EMCCD camera (Evolve). Cells were analysed either directly to visualise the cell-surface staining, or following addition of Trypan Blue (0.1% final concentration), an efficient quencher of all extracellular fluorescence (and that of permeabilised cells), to visualise the intracellular staining (supplementary material Fig. S9).

### Flow cytometry

Cells were seeded at 3×10^5^ cells per well and transfected 24 h later. After 6 h, expression was induced with 0.5 µg/ml doxycyline for 24 h. Cells were then washed and harvested as published previously (Engling et al., 2002) before incubation with primary antibody (rabbit polyclonal anti c-myc, Sigma-Aldrich, at 1.25 µg/ml) for 45 min at 4°C, then with secondary antibody (Alexa Fluor 488-conjugated goat anti-rabbit IgG, Invitrogen, at 5 µg/ml) and a dye to detect cell death (TO-PRO 3, 1:1000, Invitrogen) for 30 min at 4°C. Samples were then analysed using a FACSCalibur cytometer (BD Biosciences). Acquisition and quantification made use of CELLQuest pro software (BD Biosciences) (supplementary material Fig. S9).

### DNA constructs and recombinant proteins

DNA constructs (details given in supplementary material Methods and Table S2) are derivatives of plasmids described previously (Maizel et al., 2002; Brunet et al., 2005; Sinha et al., 2010b; Fournier et al., 2013). His6-tagged recombinant proteins produced in bacteria were purified on HisTrap columns (GE Healthcare) and, following removal of the tag by PreScisson protease cleavage, the protein was purified again on heparin columns (GE Healthcare). For protein labelling, 100 µM of purified proteins were dialysed for 2 days (20 mM phosphate buffer, 100 mM NaCl, pH 7.5) prior to incubation with a twofold molar excess of fluorescein isothiocyanate (FITC) in carbonate buffer (50 mM NaHCO_3_/Na_2_CO_3_ pH 9.5, 100 mM NaCl) overnight at 4°C and free FITC was removed by dialysis (24 h, 4°C). The efficacy of FITC incorporation was controlled by SDS-PAGE and spectral analysis. The FITC:protein molecular ratio is controlled and ranges between 1.5 and 2 for all proteins.

### Statistical analysis

The size of the sample for each experiment is given in supplementary material Table S1. Values are expressed as means±s.e.m. or statistical errors were estimated as √*p*(1−*p*)/*n*, where *p* is the percentage of embryos exhibiting a phenotype and *n* is the total number of embryos investigated (or √1/*n* when *p*=0 or 1). Comparisons between multiple groups were performed by one-way ANOVA followed by Tukey's post-test. Comparisons between the two groups were performed by a Student's *t*-test with Welch correction when variances were unequal. Quantification of anti-En effects was done with the Mann–Whitney test. *P*<0.05 was considered to indicate statistical significance.

## Supplementary Material

Supplementary Material

## References

[DEV114181C1] Ando H., Furuta T., Tsien R. Y. and Okamoto H. (2001). Photo-mediated gene activation using caged RNA/DNA in zebrafish embryos. *Nat. Genet.* 28, 317-325. 10.1038/ng583 11479592

[DEV114181C2] Araki I. and Nakamura H. (1999). Engrailed defines the position of dorsal di-mesencephalic boundary by repressing diencephalic fate. *Development* 126, 5127-5135. 10529429 10.1242/dev.126.22.5127

[DEV114181C3] Beurdeley M., Spatazza J., Lee H. H. C., Sugiyama S., Bernard C., Di Nardo A. A., Hensch T. K. and Prochiantz A. (2012). Otx2 binding to perineuronal nets persistently regulates plasticity in the mature visual cortex. *J. Neurosci.* 32, 9429-9437. 10.1523/JNEUROSCI.0394-12.2012 22764251 PMC3419577

[DEV114181C4] Brunet I., Weinl C., Piper M., Trembleau A., Volovitch M., Harris W., Prochiantz A. and Holt C. (2005). The transcription factor Engrailed-2 guides retinal axons. *Nature* 438, 94-98. 10.1038/nature04110 16267555 PMC3785142

[DEV114181C5] Chang C. P., Shen W. F., Rozenfeld S., Lawrence H. J., Largman C. and Cleary M. L. (1995). Pbx proteins display hexapeptide-dependent cooperative DNA binding with a subset of Hox proteins. *Genes Dev.* 9, 663-674. 10.1101/gad.9.6.663 7729685

[DEV114181C6] Delval S., Taminiau A., Lamy J., Lallemand C., Gilles C., Noël A. and Rezsohazy R. (2011). The Pbx interaction motif of Hoxa1 is essential for its oncogenic activity. *PLoS ONE* 6, e25247 10.1371/journal.pone.0025247 21957483 PMC3177904

[DEV114181C7] Derossi D., Joliot A. H., Chassaing G. and Prochiantz A. (1994). The third helix of the Antennapedia homeodomain translocates through biological membranes. *J. Biol. Chem.* 269, 10444-10450. 8144628

[DEV114181C8] Di Lullo E., Haton C., Le Poupon C., Volovitch M., Joliot A., Thomas J.-L. and Prochiantz A. (2011). Paracrine Pax6 activity regulates oligodendrocyte precursor cell migration in the chick embryonic neural tube. *Development* 138, 4991-5001. 10.1242/dev.066282 22028031

[DEV114181C9] Dupont E., Prochiantz A. and Joliot A. (2007). Identification of a signal peptide for unconventional secretion. *J. Biol. Chem.* 282, 8994-9000. 10.1074/jbc.M609246200 17242404

[DEV114181C10] Engling A., Backhaus R., Stegmayer C., Zehe C., Seelenmeyer C., Kehlenbach A., Schwappach B., Wegehingel S. and Nickel W. (2002). Biosynthetic FGF-2 is targeted to non-lipid raft microdomains following translocation to the extracellular surface of CHO cells. *J. Cell Sci.* 115, 3619-3631. 10.1242/jcs.00036 12186948

[DEV114181C11] Erickson T., Scholpp S., Brand M., Moens C. B. and Waskiewicz A. J. (2007). Pbx proteins cooperate with Engrailed to pattern the midbrain-hindbrain and diencephalic-mesencephalic boundaries. *Dev. Biol.* 301, 504-517. 10.1016/j.ydbio.2006.08.022 16959235 PMC1850147

[DEV114181C12] Ericson J., Rashbass P., Schedl A., Brenner-Morton S., Kawakami A., van Heyningen V., Jessell T. M. and Briscoe J. (1997). Pax6 controls progenitor cell identity and neuronal fate in response to graded Shh signaling. *Cell* 90, 169-180. 10.1016/S0092-8674(00)80323-2 9230312

[DEV114181C13] Feil R., Wagner J., Metzger D. and Chambon P. (1997). Regulation of Cre recombinase activity by mutated estrogen receptor ligand-binding domains. *Biochem. Biophys. Res. Commun.* 237, 752-757. 10.1006/bbrc.1997.7124 9299439

[DEV114181C14] Fournier L., Gauron C., Xu L., Aujard I., Le Saux T., Gagey-Eilstein N., Maurin S., Dubruille S., Baudin J.-B., Bensimon D. et al. (2013). A blue-absorbing photolabile protecting group for in vivo chromatically orthogonal photoactivation. *ACS Chem. Biol.* 8, 1528-1536. 10.1021/cb400178m 23651265

[DEV114181C15] Gehring W. J., Qian Y. Q., Billeter M., Furukubo-Tokunaga K., Schier A. F., Resendez-Perez D., Affolter M., Otting G. and Wüthrich K. (1994). Homeodomain-DNA recognition. *Cell* 78, 211-223. 10.1016/0092-8674(94)90292-5 8044836

[DEV114181C16] Hidalgo A. (1996). The roles of engrailed. *Trends Genet.* 12, 1-4. 10.1016/0168-9525(96)81373-4 8741849

[DEV114181C17] Ho R. K. and Kimmel C. B. (1993). Commitment of cell fate in the early zebrafish embryo. *Science* 261, 109-111. 10.1126/science.8316841 8316841

[DEV114181C18] Hudry B., Remacle S., Delfini M.-C., Rezsohazy R., Graba Y. and Merabet S. (2012). Hox proteins display a common and ancestral ability to diversify their interaction mode with the PBC class cofactors. *PLoS Biol.* 10, e1001351 10.1371/journal.pbio.1001351 22745600 PMC3383740

[DEV114181C19] Joliot A., Maizel A., Rosenberg D., Trembleau A., Dupas S., Volovitch M. and Prochiantz A. (1998). Identification of a signal sequence necessary for the unconventional secretion of Engrailed homeoprotein. *Curr. Biol.* 8, 856-863. 10.1016/S0960-9822(07)00346-6 9705930

[DEV114181C20] Joyner A. L. (1996). Engrailed, Wnt and Pax genes regulate midbrain--hindbrain development. *Trends Genet.* 12, 15-20. 10.1016/0168-9525(96)81383-7 8741855

[DEV114181C21] Knoepfler P. S. and Kamps M. P. (1995). The pentapeptide motif of Hox proteins is required for cooperative DNA binding with Pbx1, physically contacts Pbx1, and enhances DNA binding by Pbx1. *Mol. Cell. Biol.* 15, 5811-5819. 7565734 10.1128/mcb.15.10.5811 PMC230833

[DEV114181C22] Layalle S., Volovitch M., Mugat B., Bonneaud N., Parmentier M.-L., Prochiantz A., Joliot A. and Maschat F. (2011). Engrailed homeoprotein acts as a signaling molecule in the developing fly. *Development* 138, 2315-2323. 10.1242/dev.057059 21558379

[DEV114181C23] Le Roux I., Joliot A. H., Bloch-Gallego E., Prochiantz A. and Volovitch M. (1993). Neurotrophic activity of the Antennapedia homeodomain depends on its specific DNA-binding properties. *Proc. Natl. Acad. Sci. USA* 90, 9120-9124. 10.1073/pnas.90.19.9120 8105471 PMC47513

[DEV114181C24] Lesaffre B., Joliot A., Prochiantz A. and Volovitch M. (2007). Direct non-cell autonomous Pax6 activity regulates eye development in the zebrafish. *Neural Dev.* 2, 2 10.1186/1749-8104-2-2 17229313 PMC1797170

[DEV114181C25] Liu A. and Joyner A. L. (2001). EN and GBX2 play essential roles downstream of FGF8 in patterning the mouse mid/hindbrain region. *Development* 128, 181-191. 11124114 10.1242/dev.128.2.181

[DEV114181C26] Logan C., Wizenmann A., Drescher U., Monschau B., Bonhoeffer F. and Lumsden A. (1996). Rostral optic tectum acquires caudal characteristics following ectopic engrailed expression. *Curr. Biol.* 6, 1006-1014. 10.1016/S0960-9822(02)00645-0 8805331

[DEV114181C27] Lun K. and Brand M. (1998). A series of no isthmus (noi) alleles of the zebrafish pax2.1 gene reveals multiple signaling events in development of the midbrain-hindbrain boundary. *Development* 125, 3049-3062. 9671579 10.1242/dev.125.16.3049

[DEV114181C28] Maizel A., Bensaude O., Prochiantz A. and Joliot A. (1999). A short region of its homeodomain is necessary for engrailed nuclear export and secretion. *Development* 126, 3183-3190. 10375508 10.1242/dev.126.14.3183

[DEV114181C29] Maizel A., Tassetto M., Filhol O., Cochet C., Prochiantz A. and Joliot A. (2002). Engrailed homeoprotein secretion is a regulated process. *Development* 129, 3545-3553. 12117805 10.1242/dev.129.15.3545

[DEV114181C52] Moens C. B. and Selleri L. (2006). Hox cofactors in vertebrate development. *Dev. Biol.* 291, 193-206. 10.1016/j.ydbio.2005.10.032 16515781

[DEV114181C30] Montesinos M. L., Foucher I., Conradt M., Mainguy G., Robel L., Prochiantz A. and Volovitch M. (2001). The neuronal microtubule-associated protein 1B is under homeoprotein transcriptional control. *J. Neurosci.* 21, 3350-3359. 11331364 10.1523/JNEUROSCI.21-10-03350.2001 PMC6762475

[DEV114181C31] Morgan R., Pirard P. M., Shears L., Sohal J., Pettengell R. and Pandha H. S. (2007). Antagonism of HOX/PBX dimer formation blocks the in vivo proliferation of melanoma. *Cancer Res.* 67, 5806-5813. 10.1158/0008-5472.CAN-06-4231 17575148

[DEV114181C32] Neuteboom S. T., Peltenburg L. T., van Dijk M. A. and Murre C. (1995). The hexapeptide LFPWMR in Hoxb-8 is required for cooperative DNA binding with Pbx1 and Pbx2 proteins. *Proc. Natl. Acad. Sci. USA* 92, 9166-9170. 10.1073/pnas.92.20.9166 7568094 PMC40945

[DEV114181C33] Patel N. H., Martin-Blanco E., Coleman K. G., Poole S. J., Ellis M. C., Kornberg T. B. and Goodman C. S. (1989). Expression of engrailed proteins in arthropods, annelids, and chordates. *Cell* 58, 955-968. 10.1016/0092-8674(89)90947-1 2570637

[DEV114181C34] Peltenburg L. T. and Murre C. (1996). Engrailed and Hox homeodomain proteins contain a related Pbx interaction motif that recognizes a common structure present in Pbx. *EMBO J.* 15, 3385-3393. 8698039 PMC451902

[DEV114181C35] Phelan M. L., Rambaldi I. and Featherstone M. S. (1995). Cooperative interactions between HOX and PBX proteins mediated by a conserved peptide motif. *Mol. Cell. Biol.* 15, 3989-3997. 7623795 10.1128/mcb.15.8.3989 PMC230638

[DEV114181C36] Postlethwait J. H., Yan Y.-L., Gates M. A., Horne S., Amores A., Brownlie A., Donovan A., Egan E. S., Force A., Gong Z. et al. (1998). Vertebrate genome evolution and the zebrafish gene map. *Nat. Genet.* 18, 345-349. 10.1038/ng0498-345 9537416

[DEV114181C37] Prochiantz A. and Joliot A. (2003). Can transcription factors function as cell-cell signalling molecules? *Nat. Rev. Mol. Cell Biol.* 4, 814-819. 10.1038/nrm1227 14570063

[DEV114181C38] Radisky D. C., Stallings-Mann M., Hirai Y. and Bissell M. J. (2009). Single proteins might have dual but related functions in intracellular and extracellular microenvironments. *Nat. Rev. Mol. Cell Biol.* 10, 228-234. 10.1038/nrm2633 19190671 PMC2746016

[DEV114181C39] Scholpp S. and Brand M. (2001). Morpholino-induced knockdown of zebrafish engrailed genes eng2 and eng3 reveals redundant and unique functions in midbrain-hindbrain boundary development. *Genesis* 30, 129-133. 10.1002/gene.1047 11477690

[DEV114181C40] Scholpp S., Lohs C. and Brand M. (2003). Engrailed and Fgf8 act synergistically to maintain the boundary between diencephalon and mesencephalon. *Development* 130, 4881-4893. 10.1242/dev.00683 12917294

[DEV114181C41] Sinha D. K., Neveu P., Gagey N., Aujard I., Benbrahim-Bouzidi C., Le Saux T., Rampon C., Gauron C., Goetz B., Dubruille S. et al. (2010a). Photocontrol of protein activity in cultured cells and zebrafish with one- and two-photon illumination. *Chembiochem* 11, 653-663. 10.1002/cbic.201000008 20187057

[DEV114181C42] Sinha D. K., Neveu P., Gagey N., Aujard I., Le Saux T., Rampon C., Gauron C., Kawakami K., Leucht C., Bally-Cuif L. et al. (2010b). Photoactivation of the CreER T2 recombinase for conditional site-specific recombination with high spatiotemporal resolution. *Zebrafish* 7, 199-204. 10.1089/zeb.2009.0632 20441524

[DEV114181C43] Spatazza J., Di Lullo E., Joliot A., Dupont E., Moya K. L. and Prochiantz A. (2013a). Homeoprotein signaling in development, health, and disease: a shaking of dogmas offers challenges and promises from bench to bed. *Pharmacol. Rev.* 65, 90-104. 10.1124/pr.112.006577 23300132

[DEV114181C44] Spatazza J., Lee H. H. C., Di Nardo A. A., Tibaldi L., Joliot A., Hensch T. K. and Prochiantz A. (2013b). Choroid-plexus-derived Otx2 homeoprotein constrains adult cortical plasticity. *Cell Rep.* 3, 1815-1823. 10.1016/j.celrep.2013.05.014 23770240 PMC4119931

[DEV114181C45] Sugiyama S., Di Nardo A. A., Aizawa S., Matsuo I., Volovitch M., Prochiantz A. and Hensch T. K. (2008). Experience-dependent transfer of Otx2 homeoprotein into the visual cortex activates postnatal plasticity. *Cell* 134, 508-520. 10.1016/j.cell.2008.05.054 18692473

[DEV114181C46] Thisse C. and Thisse B. (2008). High-resolution in situ hybridization to whole-mount zebrafish embryos. *Nat. Protoc.* 3, 59-69. 10.1038/nprot.2007.514 18193022

[DEV114181C47] Volovitch M., le Roux I., Joliot A. H., Bloch-Gallego E. and Prochiantz A. (1993). Control of neuronal morphogenesis by homeoproteins: consequences for the making of neuronal networks. *Perspect. Dev. Neurobiol.* 1, 133-138. 7916257

[DEV114181C48] Wen L., Wei W., Gu W., Huang P., Ren X., Zhang Z., Zhu Z., Lin S. and Zhang B. (2008). Visualization of monoaminergic neurons and neurotoxicity of MPTP in live transgenic zebrafish. *Dev. Biol.* 314, 84-92. 10.1016/j.ydbio.2007.11.012 18164283

[DEV114181C49] Westerfield M. (1993). *The Zebrafish Book. A Guide for the Laboratory Use of Zebrafish Danio (Brachydanio) rerio*. Eugene, OR: University of Oregon Press.

[DEV114181C50] Wizenmann A., Brunet I., Lam J. S. Y., Sonnier L., Beurdeley M., Zarbalis K., Weisenhorn-Vogt D., Weinl C., Dwivedy A., Joliot A. et al. (2009). Extracellular Engrailed participates in the topographic guidance of retinal axons in vivo. *Neuron* 64, 355-366. 10.1016/j.neuron.2009.09.018 19914184 PMC4603356

[DEV114181C51] Zhou J., Qin L., Tien J. C.-Y., Gao L., Chen X., Wang F., Hsieh J.-T. and Xu J. (2012). Nkx3.1 functions as para-transcription factor to regulate gene expression and cell proliferation in non-cell autonomous manner. *J. Biol. Chem.* 287, 17248-17256. 10.1074/jbc.M111.336909 22465996 PMC3366845

