# Supplementary material for: Control of brain patterning by Engrailed paracrine transfer: a new function of the Pbx interaction domain
Source: Development. 2015 May 15;142(10):1840–9. doi: 10.1242/dev.114181 (PMC4440920; doi:10.1242/dev.114181)
Supplement: Supplementary Material [file supp_142_10_1840__index.html]

Supplementary Material 

# Control of brain patterning by Engrailed paracrine transfer: a new function of the Pbx interaction domain

## DEV114181 Supplementary Material

- Supplementary Material
