# Supplementary material for: Control of brain patterning by Engrailed paracrine transfer: a new function of the Pbx interaction domain
Source: Development. 2015 May 15;142(10):1840–9. doi: 10.1242/dev.114181 (PMC4440920; doi:10.1242/dev.114181)
Supplement: Supplementary Material [file supp_dev.114181_DEV114181supp.pdf]

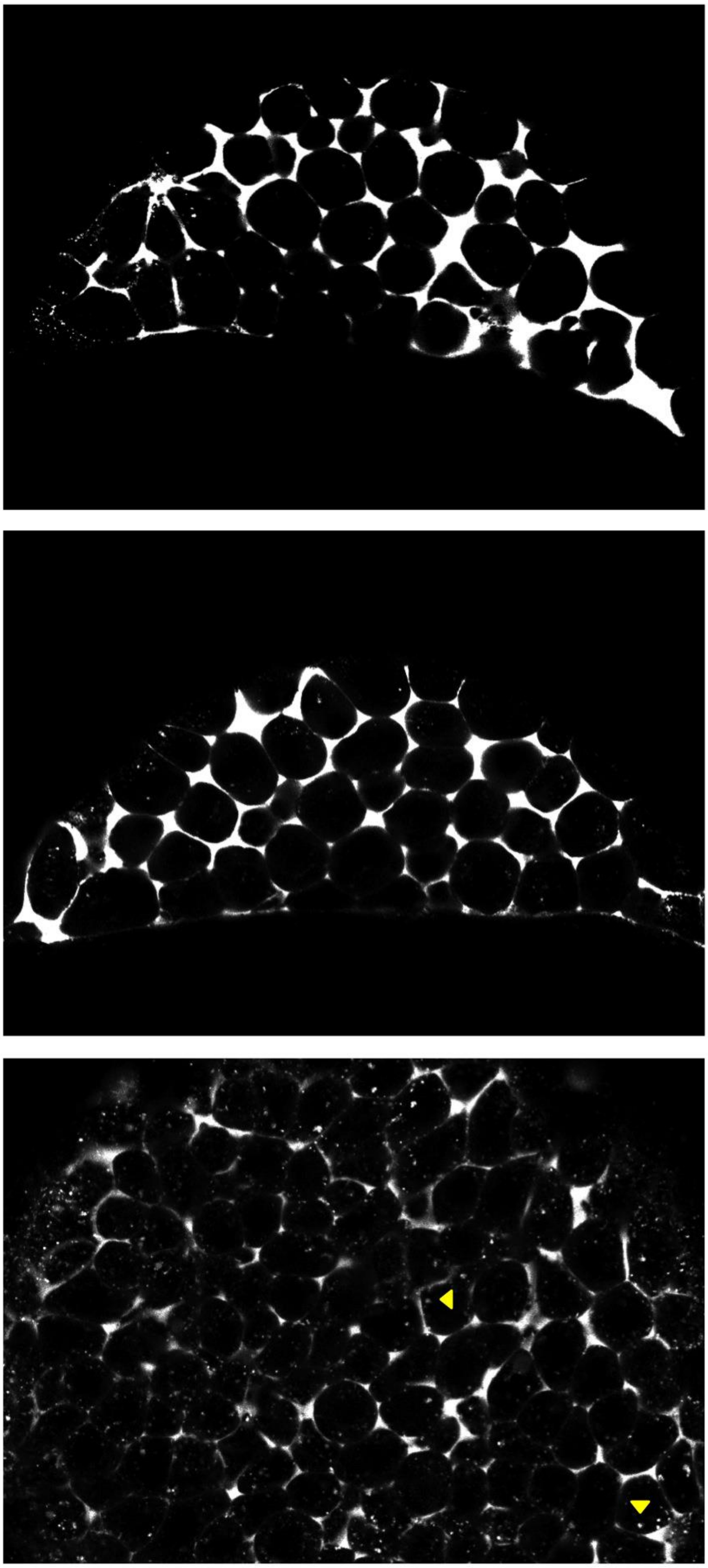

30 min post injection

60 min post injection

90 % epiboly

Figure S1: Intercellular distribution of 4G11 anti-Engrailed antibody injected at blastula stage. Embryos were injected with FITC-labeled 4G11 antibody at blastula stage. Images were taken on living embryos, at 30 and 60 minutes post injection, and at 90% epiboly. The antibody is detected in the intercellular space and is not taken-up by cells, except for residual non-specific endocytosis (arrowheads). In no occasion 4G11 antibodies were detected in the nuclei.

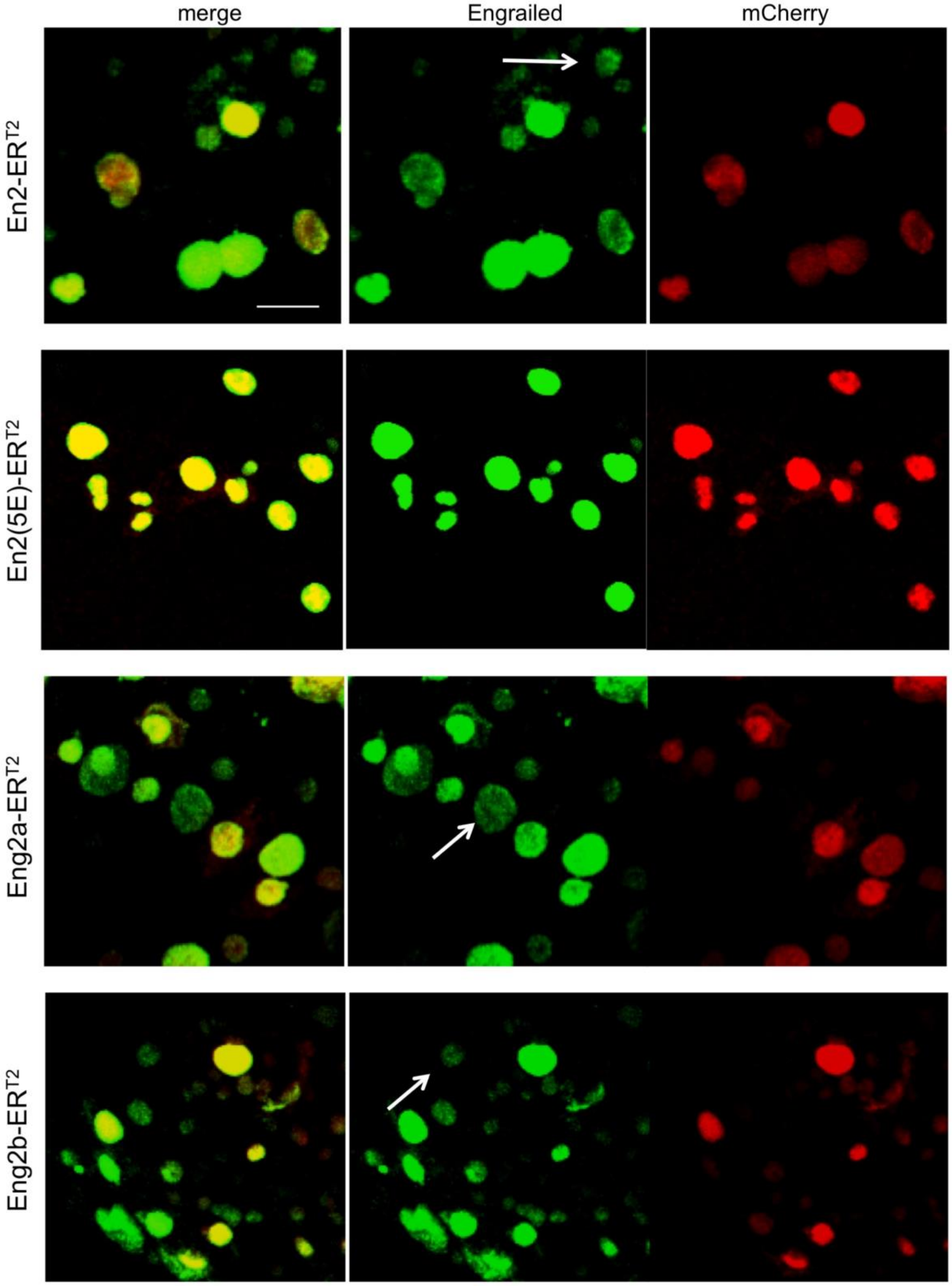

Figure S2: Intercellular transfer of En2, En25E, Eng2a and En2b in zebrafish embryos at 90% epiboly. En2-ER^{T2}-P2A-mCherry plasmid was injected at one cell stage (10 ng/ml). Embryos were fixed at 90% epiboly, and immunostained for En2ER^{T2} (Green) and mCherry (Red). Cells that do not contain the plasmid and in which Engrailed-ER^{T2} protein has been imported appear in green (white arrow). Scale bar: 20 µm.

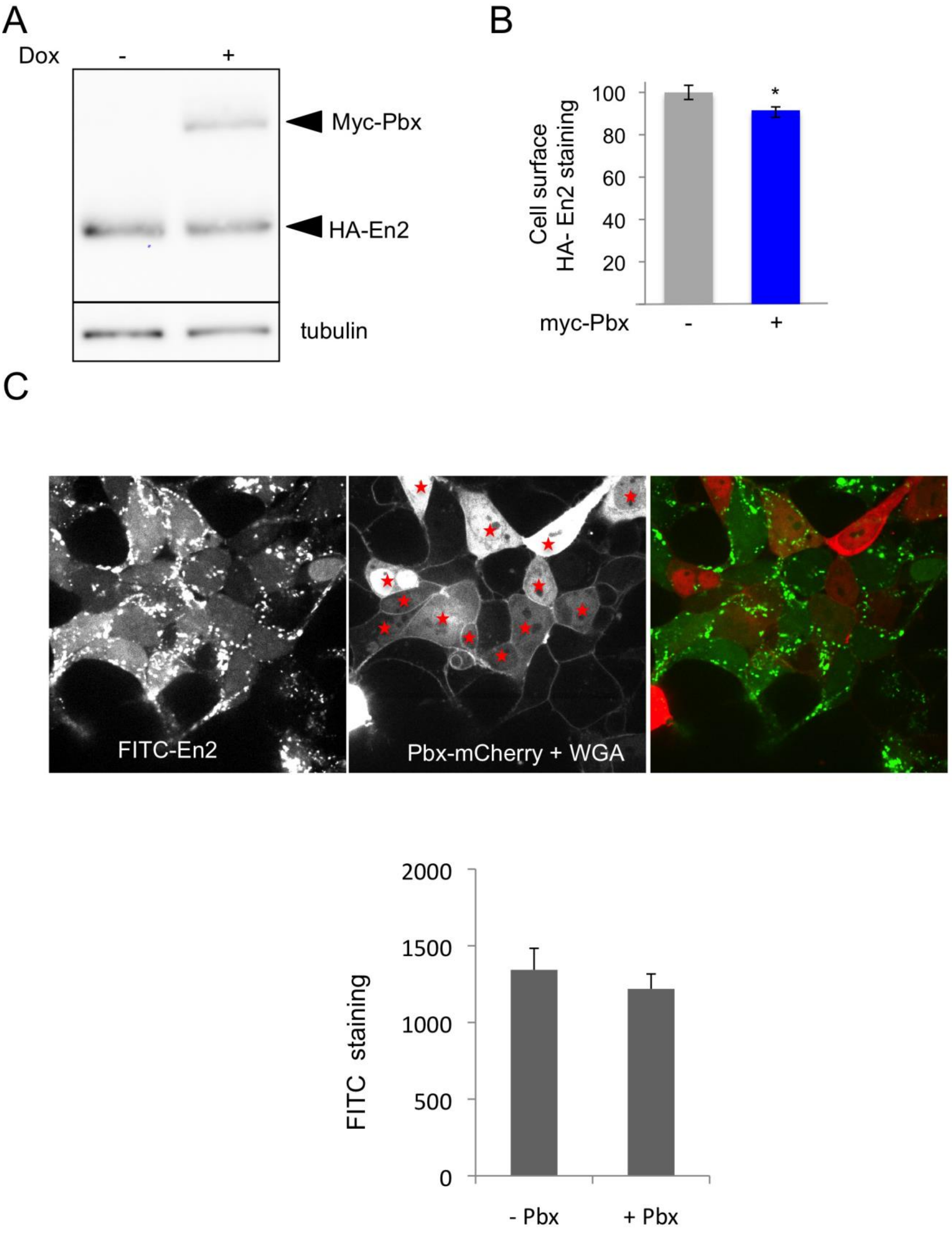

Figure S3: Sensitivity of En2 intercellular transfer to Pbx overexpression. Px1a overexpression does not affect En2 expression (**A**) and only marginally reduced En2 secretion **(B)** in HEK293 cells. **P*<0.005. (**C**) Internalization of FITC-labelled En2 in a co-culture of cells expressing Pbx1a-Cherry (red star) or not. En2 internalization quantified in each cell population is not altered by Pbx overexpression. Cherry and WGA signal are detected in the same channel.

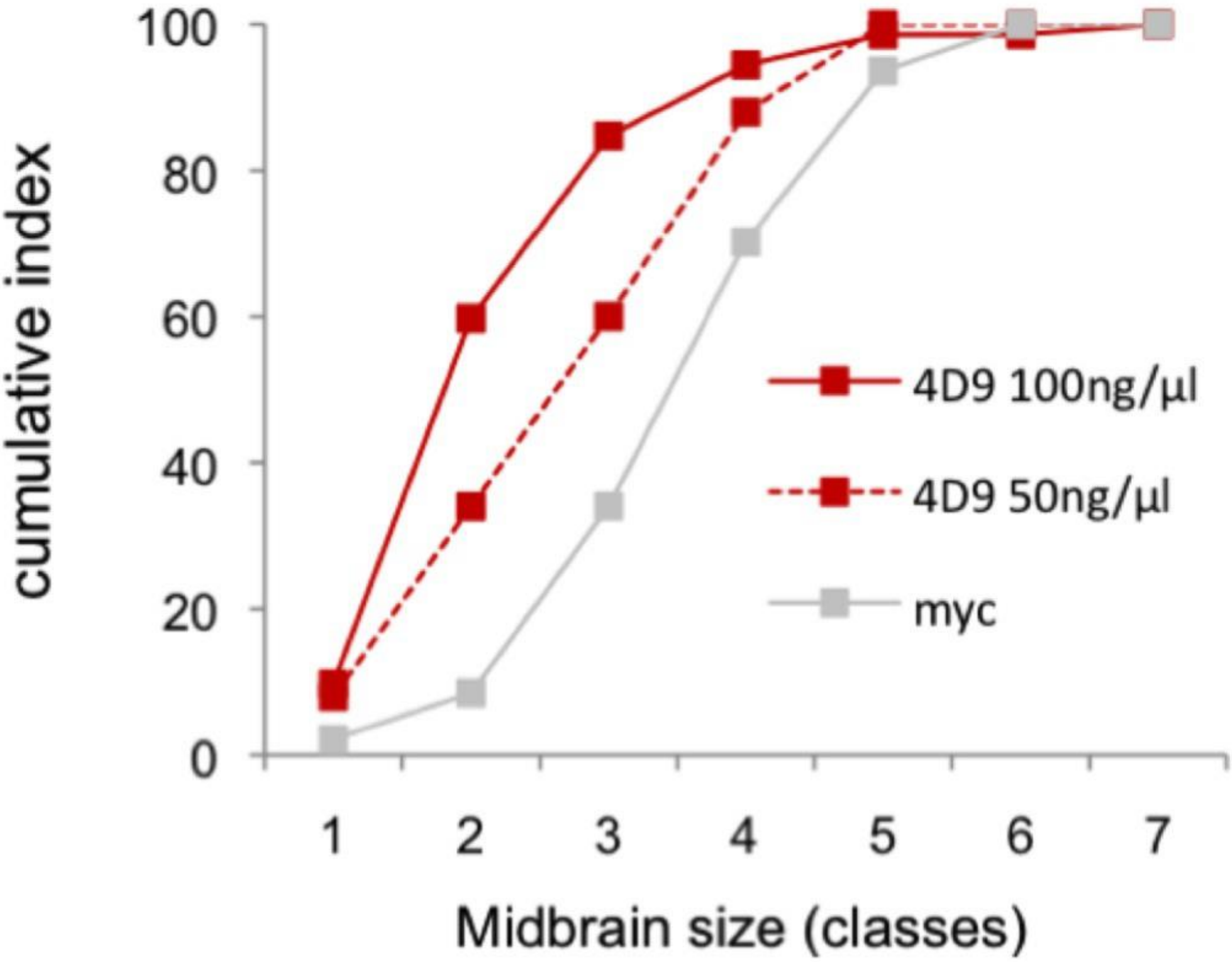

Figure S4: Dose response phenotype for 4D9 injection. Zebrafish embryos were injected intercellularly at blastula stage with anti-myc or anti-En (4D9 at 50 or 100ng/µl) antibody. Mesencephalon length of injected embryos is presented as cumulative frequency plot.

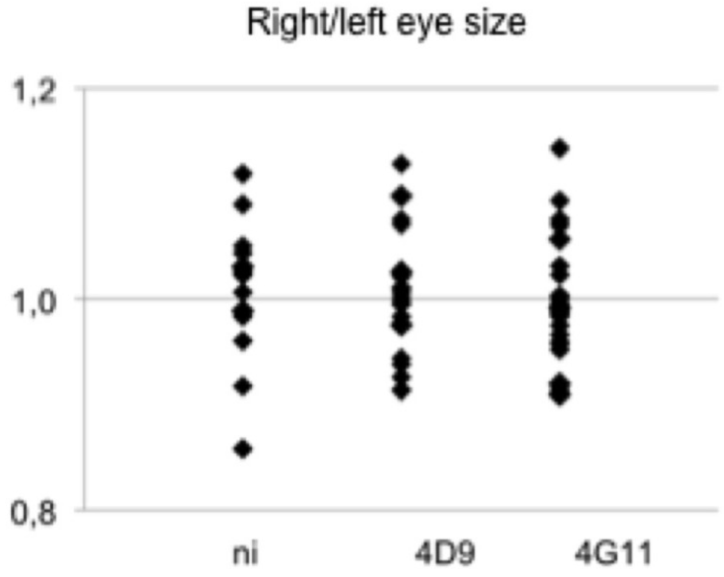

Figure S5: Absence of effect of 4D9 and 4G11 injection on eye size. Zebrafish embryos were injected intercellularly at blastula stage with anti-myc or anti-En (4G11 or 4D9) antibody (100ng/µl). Size ratios right/left eyes were measured on flat-mount embryos at 24 hpf after *pax6* ISH.

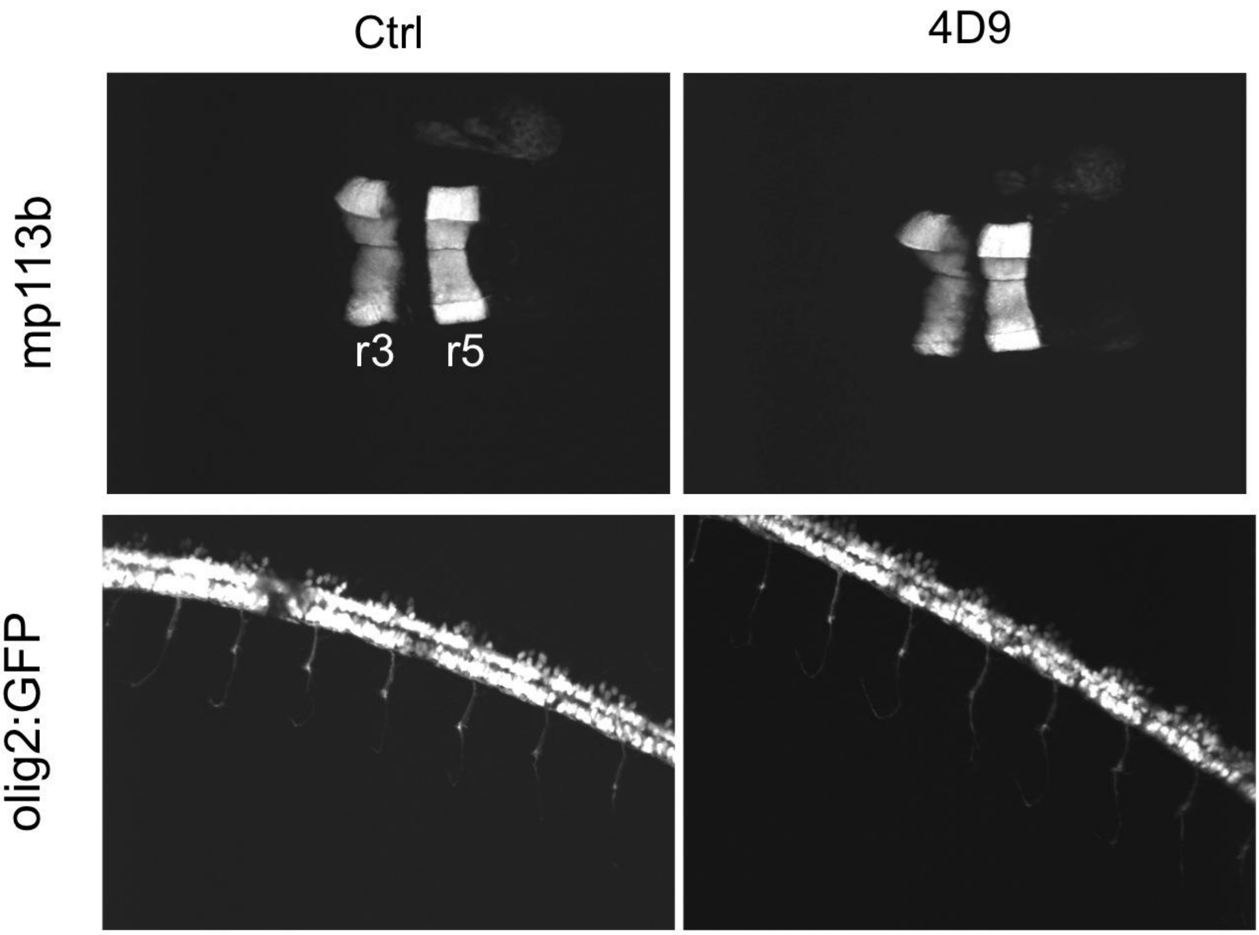

Figure S6: 4D9 injection has no effect on hindbrain (mp311b) and spinal cord (olig2) organization. Tg(mp311 +/+GFP) (Xu et al., 2012, *Development* 139(18): 3355-62), or olig2:gfp Tg(*olig2*:EGFP) (Shin et al., 2003, *Methods Cell Sci* 25(1-2): 7-14), transgenic zebrafish embryos were injected intercellularly at blastula stage with anti-myc or anti-En (4D9) antibody. Phenotype observed at 24 hpf. Dorsal view (mp311b) lateral view (olig2). r = rhombomere. Inhibition of Engrailed intercellular transfer has no effect on rhombomere development or oligodendrocyte migration.

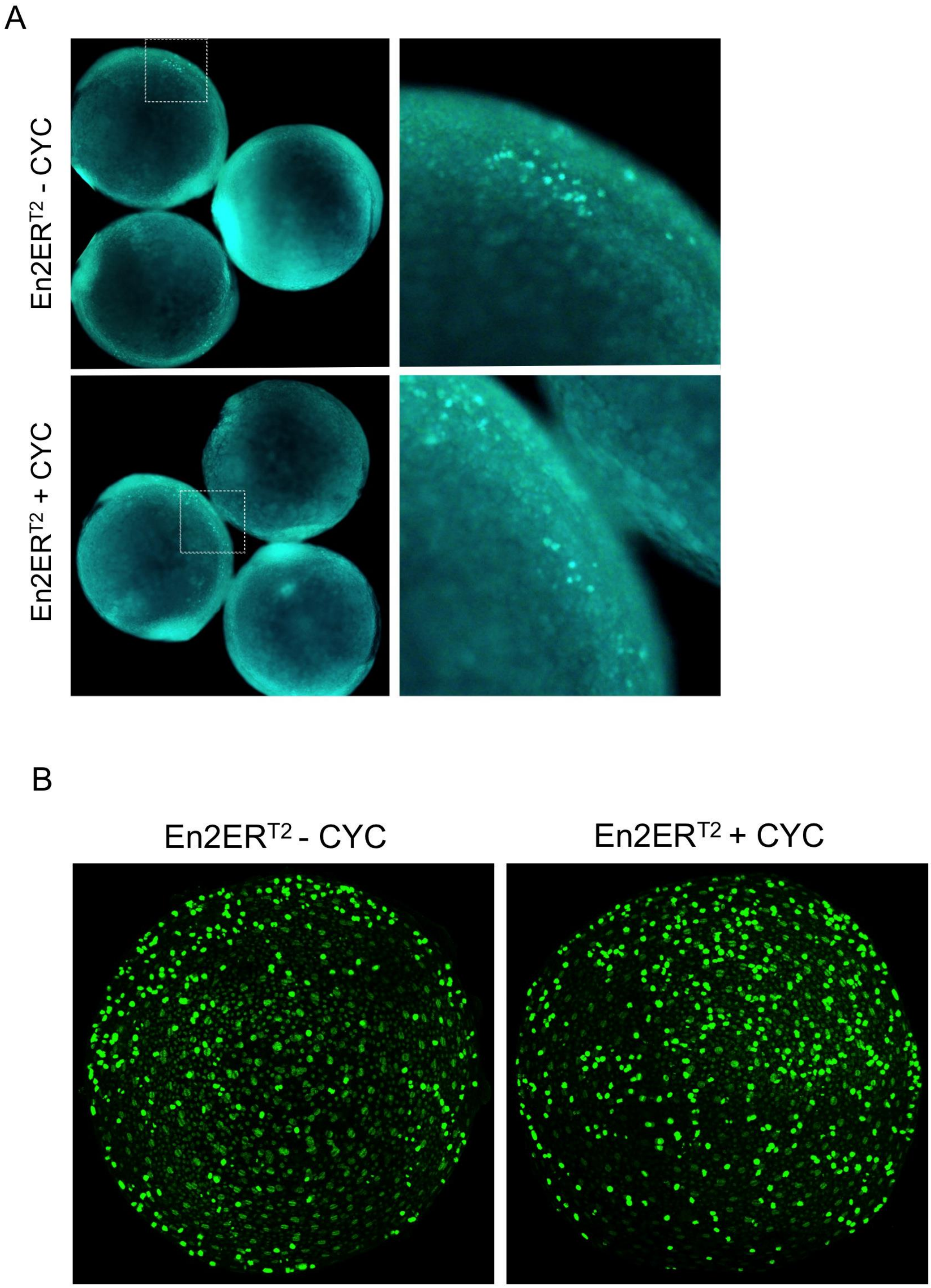

Figure S7: Proliferation and apoptosis are not affected by En2 gain of function. mRNA encoding En2ERT2 was injected at one-cell stage, incubated in caged CYC and the protein activated at 70% epiboly. Embryos were either stained with acridine orange to visualize dying cells (**A**) or fixed at 90% epiboly for P-H3 immunostaining to detect cell in mitosis (**B**). No significant difference was observed.

4G11 Epitope

Chick En1	TNFFIDNILRPDFG
Chick En2	TNFFIDNILRPEFG
Danio Eng2a	TNFFIDNILRPDFG
Danio Eng2b	TNFYIDNILRPDFG

4D9 Epitope

Droso En	ELGLNESQIKIWF
Chick En2	ELGLNESQIKIWF
Danio Eng2a	ELGLNESQIKIWF
Danio Eng2b	ELGLNESQIKIWF

Figure S8: Conservation 4D9 and 4G11 epitopes in the studied Engrailed proteins. 4D9 and 4G11 epitopes are defined according (Patel et al. Cell 1989;58:955–968) and (Ericson et al. Cell 1997;90:169–180) respectively.

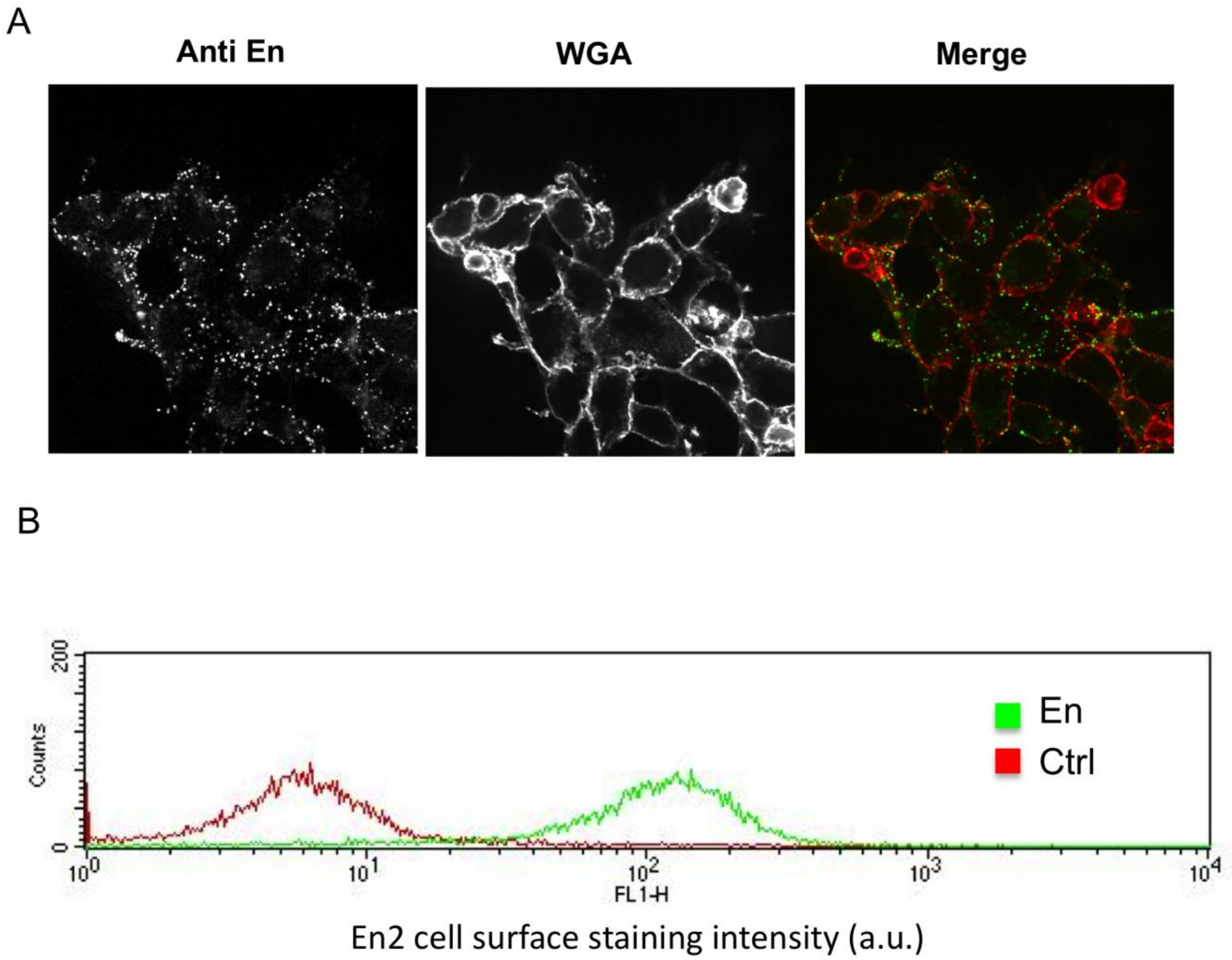

Figure S9: (**A**) Vizualisation of extracellular En2 staining. HEK293 expressing En2 were fixed and immunostained for Engrailed (4G11) and the plasma membrane marker wheat germ agglutinin (WGA) in the absence of permeabilization treatment. (**B**) Exemplary FACS profile of En surface staining in the presence (En) or absence (ctrl) Engrailed expression.

Supplementary Materials and Methods

Plasmids

Plasmids used for in vitro transcription (pCSmEn2ERT, coding for myc-En2-ER^{T2}; pCSmEn25EERT, coding for myc-En2(Ser150,151,153,155,156Glu)-ER^{T2}; pCSmEn2SRERT, coding for myc-En2(W247S,F248R)-ER^{T2}; pShaEng2aE4PCh, coding for HA-Eng2a-ER^{T2}-P2A-mCherry; pShaEng2bE4PCh, coding for HA-Eng2b-ER^{T2}-P2A-mCherry; pCSm5En2WWER4, coding for 5xmyc-En2(W169K,W172K)-ER^{T2}; pShaEng2aKRPC, coding for HA-Eng2a(W145K,W148K)-ER^{T2}-P2A-mCherry; pShaEng2bKRPC, coding for HA-Eng2b(W141K,W144K)-ER^{T2}-P2A-mCherry) were built in derivatives of pCS2 (Turner & Weintraub, 1994). Plasmid for expression in zebrafish embryos (pT22Ubm5En2E4PCh, coding for 5xmyc-En2-ER^{T2}-P2A-mCherry) contains an Ubi promoter (Mosiman et al., 2011). Plasmids for protein expression in E. coli (pShCherryScEn2, coding for bactCherry-ScissionCS-En2; pShCherryScEng2a, coding for bactCherry-ScissionCS-Eng2a; pShCherryScEng2b, coding for bactCherry-ScissionCS-Eng2b; pShCherryScEn2WW, coding for bactCherry-ScissionCS-En2(W169K,W172K) were based on CherryExpress™ vector from Eurogentec SA (Belgium). Plasmids for transfection in mammalian cells (pCSEng2a or pcDNA7Eng2a, coding for Eng2a; pCSEng2b or pcDNA7Eng2b, coding for Eng2b; pcDNA7En2, coding for En2; pcDNA7En2K, coding for En2(W169K,W172K); pcDNA7Eng2aK, coding for Eng2a(W145K,W148K); pcDNA7Eng2bK, coding for Eng2b(W141K,W144K)) were built in derivatives of either pCS2 or pcDNA5 (Invitrogen, Thermo Fisher Scientific, USA). Sequences are available on request.

Table S1: Sample size

**Figure 1B:** no CYC[n=33]; dome[n=44]; 50% epiboly[n=18]; 70% epiboly[n=20]; 1-2 somite[n=13].<br><br>**Figure 1D**: Ctrl[n=54; En2 gain-of-function[n=66]
**Figure 2B:** no CYC, 5E[n=78]; no CYC, SR[n=29]; dome, 5E[n=16]; dome, SR[n=57]; 50% epiboly, 5E[n=78]; 50% epiboly, SR[n=41; 70% epiboly, 5E[n=21]<br><br>**Figure 2C**: Ctrl[n=6], En2[n=6], En-5E[n=6],<br><br>**Figure 2E:** EnER^{T2} no CYC[n=18]; EnER^{T2} + CYC[n=57]; EnER^{T2} + CYC+ 4G11[n=136]; EnER^{T2} + CYC+ 4D9[n=29]; 4G11[n=48]; 4D9[n=79]
**Figure 3D:** En2[n= 14 samples*]; Eng2a[n=15 samples*]; Eng2b[n=14 samples*]<br><br>**Figure 3E:** For each condition 8 independent samples of 10,000 cells were used for quantification.<br><br>**Figure 3F:** EnER^{T2} no CYC[n=47]; EnER^{T2} + CYC[n=31]; Eng2aER^{T2} no CYC[n=42]; Eng2aER^{T2} + CYC[n=43]; Eng2bER^{T2} no CYC[n=81]; Eng2bER^{T2} + CYC[n=101]
**Figure 4E:** anti-myc[n=47]; 4D9[n=72]; 4D9+peptide[n=26];<br><br>**Figure 4F:** anti-myc[n=37]; 4D9[n=59]; 4D9+peptide[n=37];<br><br>**Figure 4G**: anti-myc[n=47]; 4G11[n=54];<br><br>**Figure 4H:** anti-myc[n=37]; 4G11[n=23];<br><br>**Figure 4I**: En2[n=80], En2+4D9[n=60], Eng2a[n=49], En2a+4D9[n=45], Eng2b[n=59], Eng2b+4D9[n=38];<br><br>**Figure 4J:** Ctrl[n=6], En2[n=6], En2a[n=6], En2b[n=6],
**Figure 5:** En2 gain-of-function[n=66]; Ctrl[n=54]; anti-myc[n=23]; anti En2[n=60]
**Figure 6A**: En2[n=52]; En2WW>KK[n=42]; Eng2a[n=56]; Eng2aWW>KK[n=76]; Eng2b[n=31]; Eng2bWW>KK[n=72]<br><br>**Figure 6B**: For each condition 8 independent samples of 10,000 cells were used for quantification.<br><br>**Figure 6D:** En2[n= 8 samples*]; En2WW>KK[n=8 samples*]<br><br>**Figure 6F:** En2[n= 8 samples*]; HD[n= 8 samples*]; Hexa-HD[n= 8 samples*]
**Figure S3B:** For each condition 8 independent samples of 10,000 cells were used for quantification.<br>**Figure S3C:** [n= 8 samples*]
**Figure S4:** anti-myc[n=47]; 4D9 50 ng/ml[n=50]; 4D9 100ng/ml[n=72];
**Figure S5:** ni[n=38]; 4D9[n=42]; 4G11[n=44]

* for each sample the fluorescence of at least 10 cells was measured

Table S2. Plasmids used in this study

Plasmid	Promoter	Coding sequence
pCSm5En2ERT	SP6	5xmyc-chkEn2-ER^{T2}
pCSmEn25EERT	SP6	myc-chkEn2(5S>5E)-ER^{T2}
pCSmEn2SRERT	SP6	myc-chkEn2(WF>SR)-ER^{T2}
pShaEng2aE4PCh	SP6	3xHA-Eng2a-ER^{T2}-P2A-mCherry
pShaEng2bE4PCh	SP6	3xHA-Eng2b-ER^{T2}-P2A-mCherry
pCSm5En2WWER4	SP6	myc-chkEn2(WW>KK)- ER^{T2}
pShaEng2aKRPC	SP6	3xHA-Eng2a(WW>KK)-ER^{T2}-P2A-mCherry
pShaEng2bKRPC	SP6	3xHA-Eng2b(WW>KK)-ER^{T2}-P2A-mCherry
pT22Ubm5En2E4PCh	Ubi	5xmyc-chkEn2-ER^{T2}-P2A-mCherry
pShCherryScEn2	T7	bacCherry-Sci-chkEn2
pShCherryScEng2a	T7	bacCherry-Sci-Eng2a
pShCherryScEng2a	T7	bacCherry-Sci-Eng2b
pShCherryScEn2WW	T7	bacCherry-Sci-chkEn2(WW>KK)
pCSEng2a	sCMV	Eng2a
pCSEng2b	sCMV	Eng2b
pcDNA7En2	CMV	chkEn2
pcDNA7Eng2a	CMV	Eng2a
pcDNA7Eng2b	CMV	Eng2b
pcDNA7En2K	CMV	chkEn2(WW>KK)
pcDNA7Eng2aK	CMV	Eng2a(WW>KK)
pcDNA7Eng2bK	CMV	Eng2b(WW>KK)

Sequences are available on request.
